# Supplementary material for: Integrated omics approach to unveil antifungal bacterial polyynes as acetyl-CoA acetyltransferase inhibitors
Source: Commun Biol. 2022 May 12;5:454. doi: 10.1038/s42003-022-03409-6 (PMC9098870; doi:10.1038/s42003-022-03409-6)
Supplement: Supplementary file 2 — Supplementary Information [file 42003_2022_3409_MOESM2_ESM.pdf]

Supplementary Information

Integrated omics approach to unveil antifungal bacterial polyynes as acetyl-CoA acetyltransferase inhibitors

Ching-Chih Lin[1,2,#], Sin Yong Hoo[1,2,#], Li-Ting Ma[1,2,#], Chih Lin[1], Kai-Fa Huang[3], Ying-Ning Ho[4],Chi-Hui Sun[1], Han-Jung Lee[1], Pi-Yu Chen[1], Lin-Jie Shu[1], Bo-Wei Wang[1,2,5], Wei-Chen Hsu[1,2], Tzu-Ping Ko[3], and Yu-Liang Yang[1,2,*]

[1] Agricultural Biotechnology Research Center, Academia Sinica, Nankang Dist., Taipei 115, Taiwan
[2] Biotechnology Center in Southern Taiwan, Academia Sinica, Guiren Dist., Tainan 711, Taiwan
[3] Institute of Biological Chemistry, Academia Sinica, Nankang Dist., Taipei 115, Taiwan
[4] Institute of Marine Biology and Center of Excellence for the Oceans, National Taiwan Ocean University, Jhongjheng Dist., Keelung 202, Taiwan
[5] Department of Marine Biotechnology and Resources, National Sun Yat-sen University, Gushan Dist., Kaohsiung 804, Taiwan

[#]These authors contributed equally.
*Corresponding author: Dr. Yu-Liang Yang
Phone number: +886-6-3032836
E-mail: ylyang@gate.sinica.edu.tw

Contents	Title	Page
**Supplementary Figures**		
**Figure 1**	Discriminated antifungal phenotype and differential expression of biosynthetic gene clusters of *Massilia* sp. YMA4	4
**Figure 2**	Construction scheme (a) and PCR check results (b) of null mutant strains YMA4::*masD*, YMA4::*masE*, YMA4::*masF*, YMA4::*masH,* YMA4::*masI,* YMA4::*masJ,* YMA4::*masK* and YMA4::*masL*	5
**Figure 3**	Extraction ion chromatography (EIC) of polyynes (*m/z* 257.1183 ± 10 ppm window) in *Massilia* sp. YMA4 wild type and the biosynthesis null mutant strains	6
**Figure 4**	UPLC-DAD-HRMS of dominant polyynes in *Massilia* sp. YMA4. Asterisks in EIC indicate the polyyne compounds	7
**Figure 5**	Tandem mass spectra and fragment annotation of polyynes	8
**Figure 6**	^{1}H NMR spectrum of collimonin C **1**	9
**Figure 7**	^{1}H-^{1}H COSY spectrum of collimonin C **1**	9
**Figure 8**	HSQC spectrum of collimonin C **1**	10
**Figure 9**	HMBC spectrum of collimonin C **1**	10
**Figure 10**	^{1}H NMR spectrum of collimonin D **2**	11
**Figure 11**	^{1}H-^{1}H COSY spectrum of collimonin D **2**	11
**Figure 12**	HSQC spectrum of collimonin D **2**	12
**Figure 13**	HMBC spectrum of collimonin D **2**	12
**Figure 14**	^{1}H NMR spectrum of massilin A **3**	13
**Figure 15**	^{1}H-^{1}H COSY spectrum of massilin A **3**	13
**Figure 16**	HSQC spectrum of massilin A **3**	14
**Figure 17**	HMBC spectrum of massilin A **3**	14
**Figure 18**	^{1}H NMR spectrum of massilin B **4**	15
**Figure 19**	^{1}H-^{1}H COSY spectrum of massilin B **4**	15
**Figure 20**	HSQC spectrum of massilin B **4**	16
**Figure 21**	HMBC spectrum of massilin B **4**	16
**Figure 22**	Minimum inhibitory concentration (MIC) of polyynes and clinical drug against *C. albicans* ATCC18804 in YPD medium	17
**Figure 23**	Phylogenetic analysis of bacterial polyyne BGCs	18
**Figure 24**	LC-DAD-HRMS of the biosynthetic product from *E. coli* co-expression of *mas* genes	19
**Figure 25**	^{1}H NMR spectrum of massilin C **5**	20
**Figure 26**	^{1}H-^{1}H COSY spectrum of massilin C **5**	20
**Figure 27**	HSQC spectrum of massilin C **5**	21
**Figure 28**	HMBC spectrum of massilin C **5**	21
**Figure 29**	The chemical structures of C16 and C18 polyynes annotated in Supplementary Figure 23	22
**Figure 30**	Kinetic evaluation of irreversible inhibitors and polyynes-MasL experiment detail	23
**Figure 31**	Overall structures of MasL, MasL-collimonin C, and MasL-collimonin D complex	24
**Figure 32**	Sequence alignment of acetyl-CoA acetyltransferases from different organisms	25
**Figure 33**	Electron density map of collimonin C **1** in MasL reactive pocket.	26
**Figure 34**	Electron density map of collimonin D **2** in MasL reactive pocket	27
**Figure 35**	Bottom-up proteomics analysis of MasL treated by polyynes	28
**Figure 36**	Magnification view of MasL covalently modified by collimonin C/D **1**, **2**	29
**Figure 37**	Residual enzyme activity of *C. albicans* ERG10 treated by polyynes	30
**Figure 38**	Bottom-up proteomics analysis of *C. albicans* ERG10 treated by polyynes	31

**Figure 39**	Transmission electron microscopy (TEM) images of polyyne-treated *C. albicans* ATCC18804	32
**Figure 40**	LC-HRMS/MS profiles and tandem mass spectra of polyynes from *B. vietnamiensis* LMG 10929 and *Massilia* sp. YMA4	33
**Figure 41**	Tetracycline-inducible expression system in *C. albicans* ATCC18804.	34
**Supplementary Tables**		
**Table 1**	^{1}H and ^{13}C NMR of collimonin C **1** in C_2D_6OS	35
**Table 2**	^{1}H and ^{13}C NMR of collimonin D **2** in C_2D_6OS	36
**Table 3**	^{1}H and ^{13}C NMR of massilin A **3** in C_2D_6OS	37
**Table 4**	^{1}H and ^{13}C NMR of massilin B **4** in C_2D_6OS	38
**Table 5**	^{1}H and ^{13}C NMR of massilin C **5** in C_2D_6OS	49
**Table 6**	Characterization of *mas* BGC and core genes in *ccn* BGC	40
**Table 7**	The cell viabilities of *C. albicans* OE *masL* and OE *ERG10* under various polyynes.	41
**Table 8**	List of primer sets used in this study	42
**Table 9**	List of plasmids used in this study.	44
**Table 10**	List of strains used in this study	45
**Supplementary Methods**		
Chemicals, strains, plasmids, and culture conditions		46
RNA sequencing and transcriptomic analysis		46
Construction of polyyne biosynthesis gene-null mutant strains		46
UPLC-DAD-MS/MS methods		47
Construction of *mas* co-expression platform in *E. coli*		47
Isolation, structure elucidation, and quantification of polyynes in *Massilia* sp. YMA4		47
Construction of inducible *ERG10* overexpression strains and $masL_{opt}$ heterologous expression strain in *C. albicans*		49
Transmission Electron Microscope		50
Construction of inducible *masL* heterologous expression strain in *E. coli*		50
Expression and purification of MasL and ERG10		50
Enzymatic inhibition assay and inhibition kinetics of polyynes		51
Protein crystallization, data collection, processing, and refinement		51
Sequence alignment and structure superimposition		52
Bottom-up mass spectrometry analysis and peptide mapping of polyyne-labeled peptides		52
**SI References**		53

Supplementary Figures

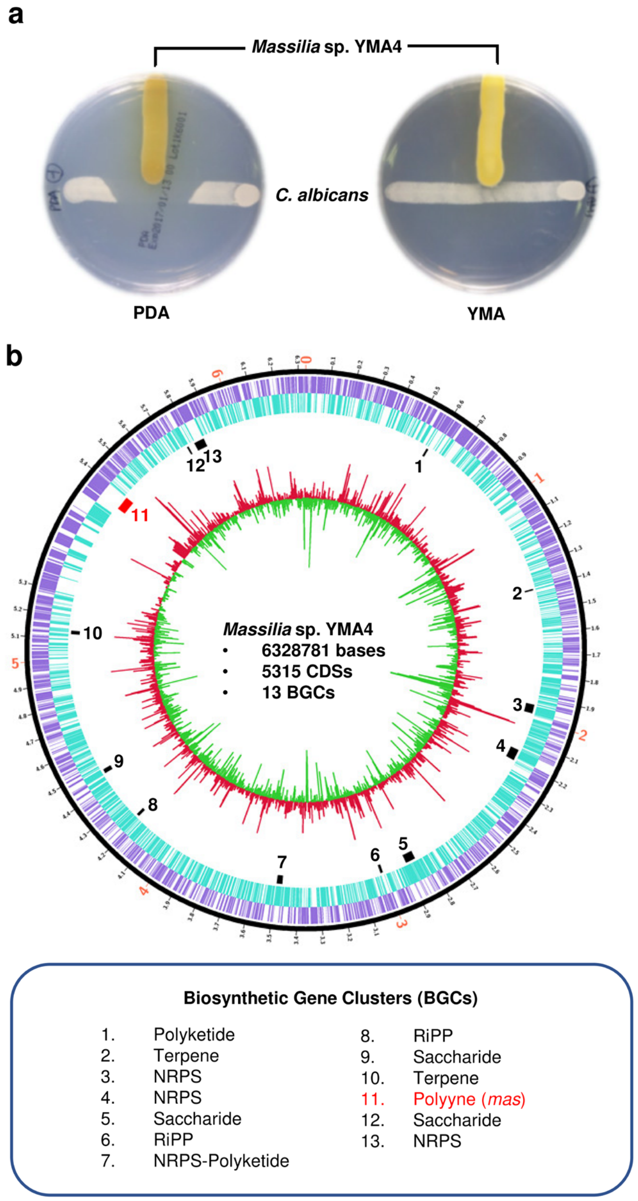

Supplementary Figure 1. Discriminated antifungal phenotype and differential expression of biosynthetic gene clusters of *Massilia* sp. YMA4. (a) Antagonism assay of *Massilia* sp. YMA4 against *C. albicans* on PDA (active) and YMA (inactive) media. **(b)** Whole-genome sequence and RNA-seq analysis of *Massilia* sp. YMA4 on PDA (active) and YMA (inactive) media. Megabases are labeled as red on the outer black track; smaller ticks correspond to 100 kbp segments. The circular track from outside to inside represent: (1) Coding sequences (CDSs) on forward strand (purple); (2) CDSs on reverse strand (blue); (3) Predicted biosynthetic gene clusters (BGCs, black and red) and polyyne BGC (red); (4) Fold change histogram of CDSs of *Massilia* sp. YMA4 on PDA versus YMA, red indicates upregulation, and green indicates downregulation. NRPS represents the nonribosomal peptide-synthetase; RiPP represents ribosomally synthesized and post-translationally modified peptides. Complete data was provided in **Supplementary Data 1** and **Supplementary Figure 23**.

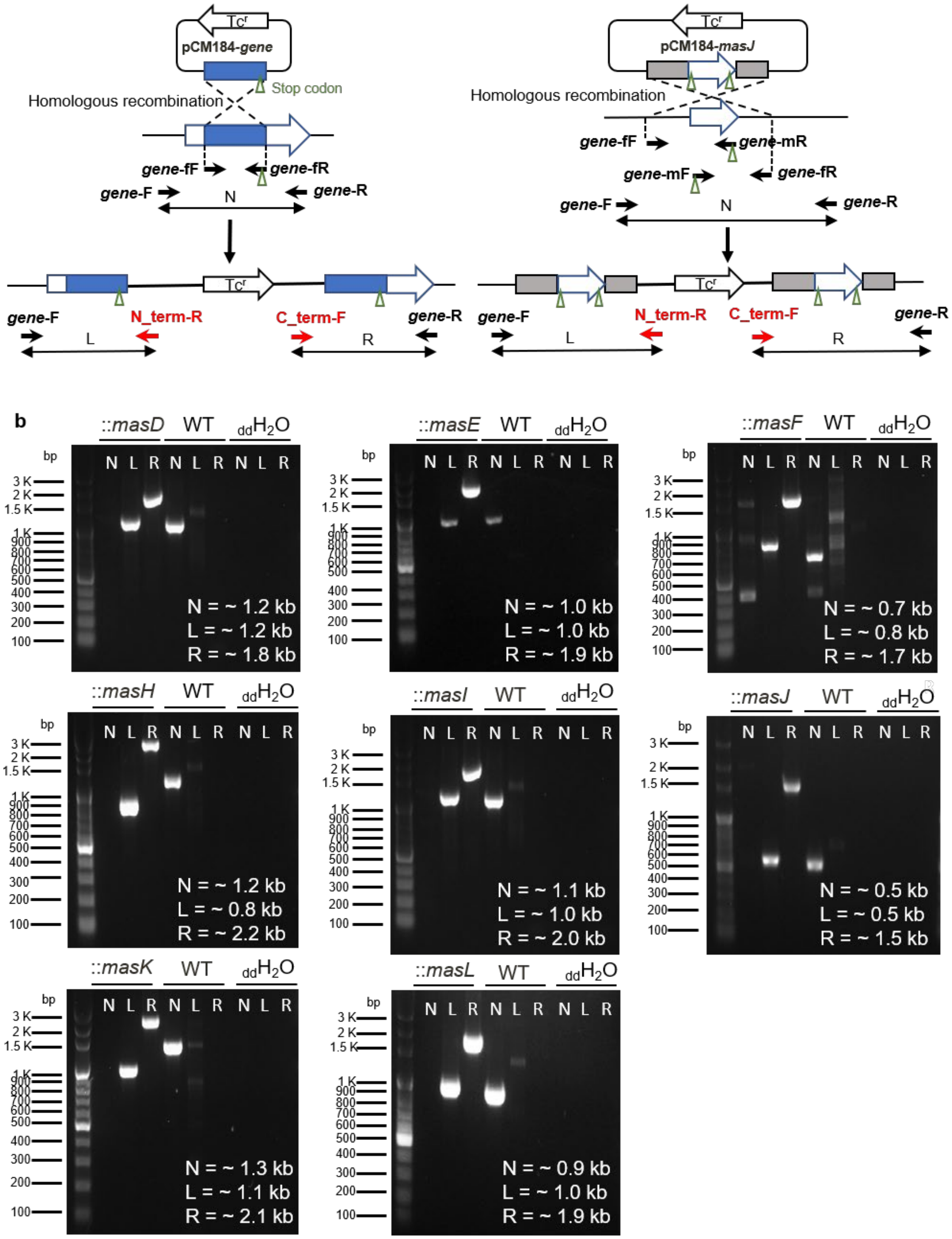

Supplementary Figure 2. Construction scheme (a) and PCR check results (b) of null mutant strains YMA4::*masD*, YMA4::*masE*, YMA4::*masF*, YMA4::*masH,* YMA4::*masI,* YMA4::*masJ,* YMA4::*masK* and YMA4::*masL*. The positions of the primer sets are shown in panel (**a**).

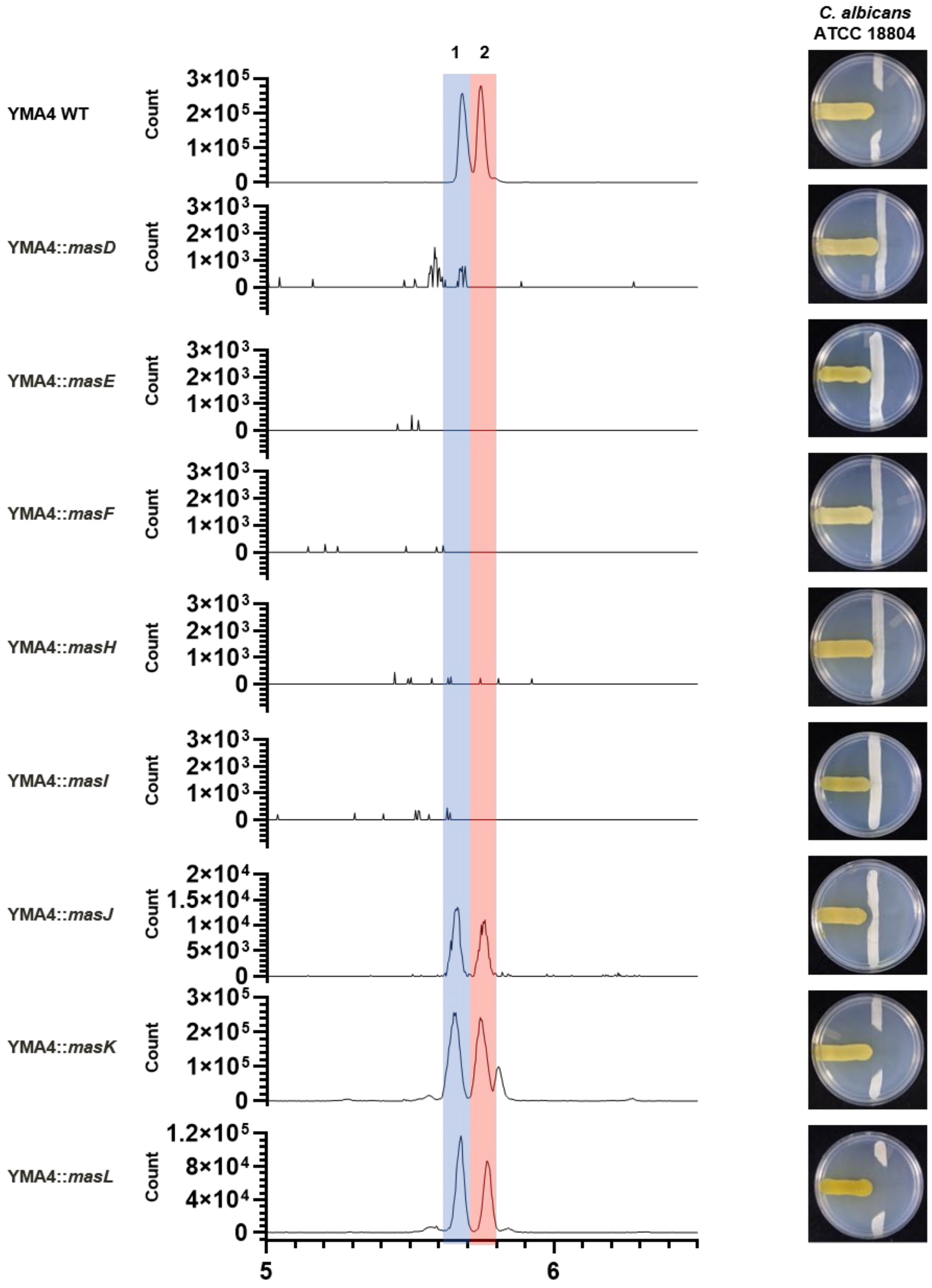

Supplementary Figure 3. Extraction ion chromatography (EIC) of polyynes (*m/z* 257.1183 ± 10 ppm window) in *Massilia* sp. YMA4 wild type and the biosynthesis null mutant strains (left panel). The colored boxes indicate collimonin C **1** (blue) and collimonin D **2** (red). The antagonism assay of *Massilia* sp. YMA4 wild type and mutants against *C. albicans* (right panel).

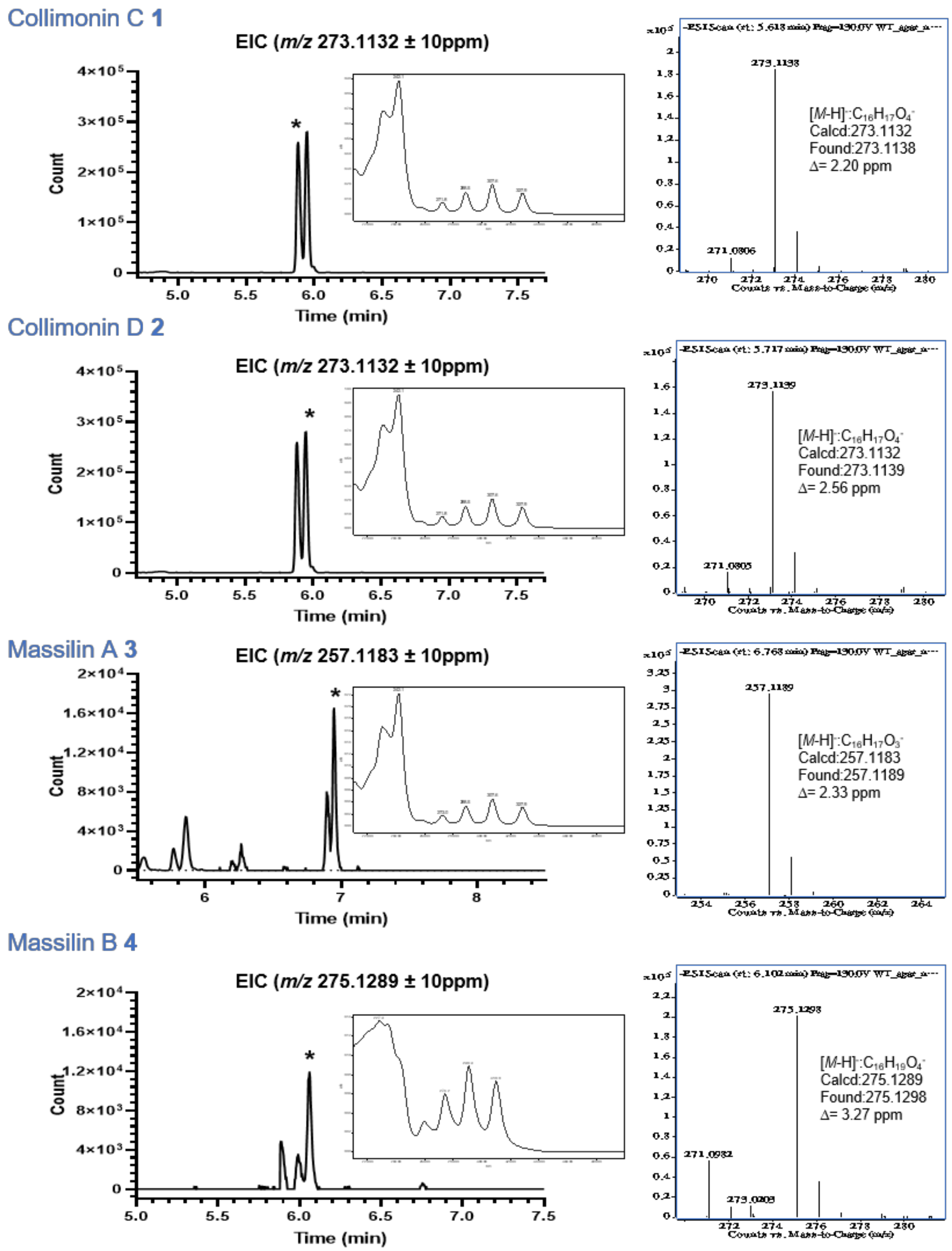

Supplementary Figure 4. UPLC-DAD-HRMS of dominant polyynes in *Massilia* sp. YMA4. Asterisks in EIC indicate the polyyne compounds.

Collimonin C 1

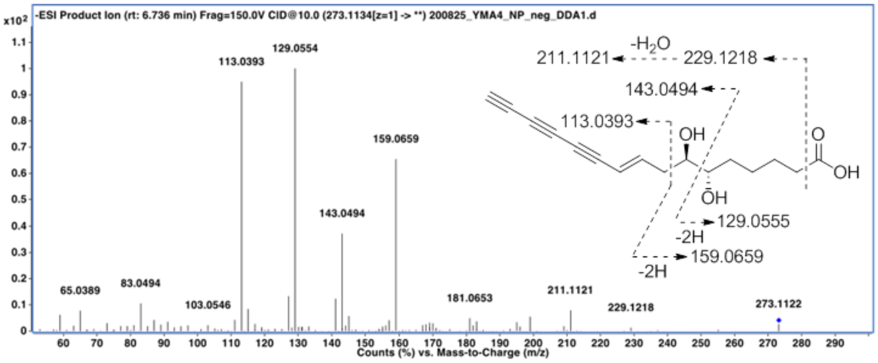

Collimonin D 2

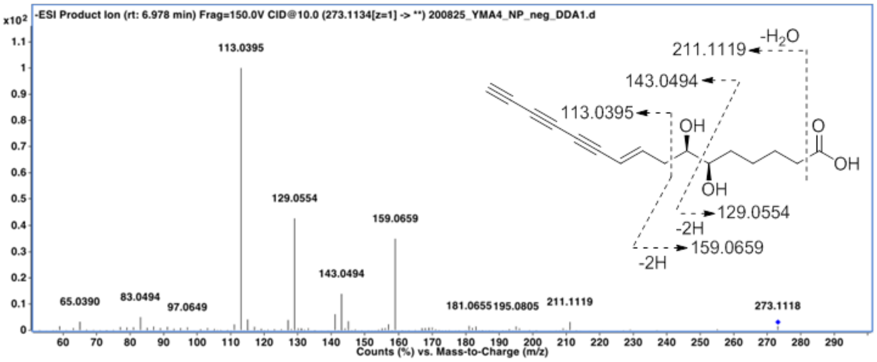

Massilin A 3

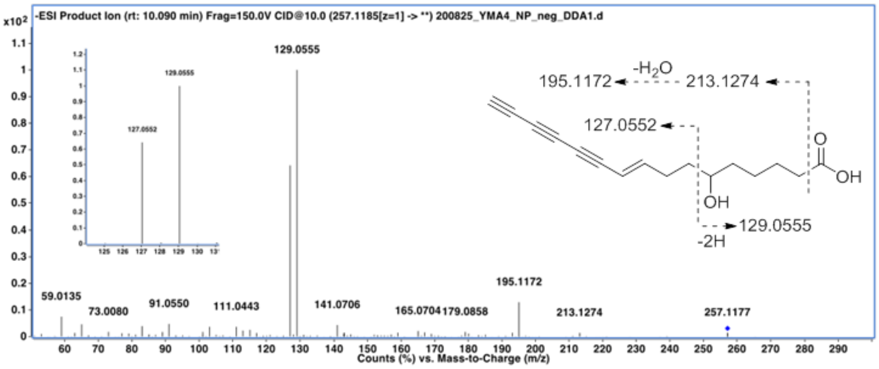

Massilin B 4

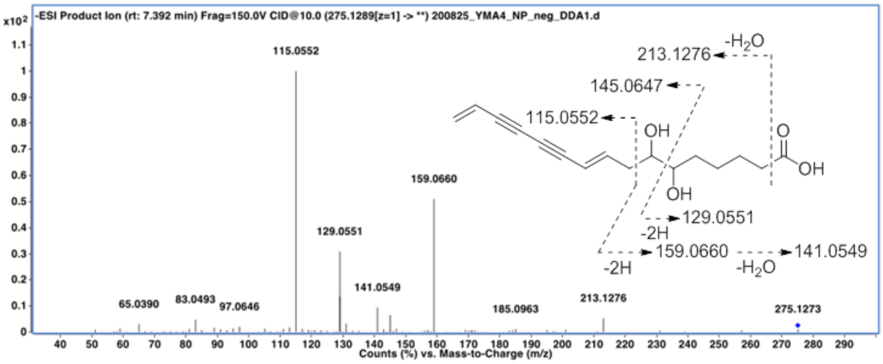

Supplementary Figure 5. Tandem mass spectra and fragment annotation of polyynes.

^{1}H NMR

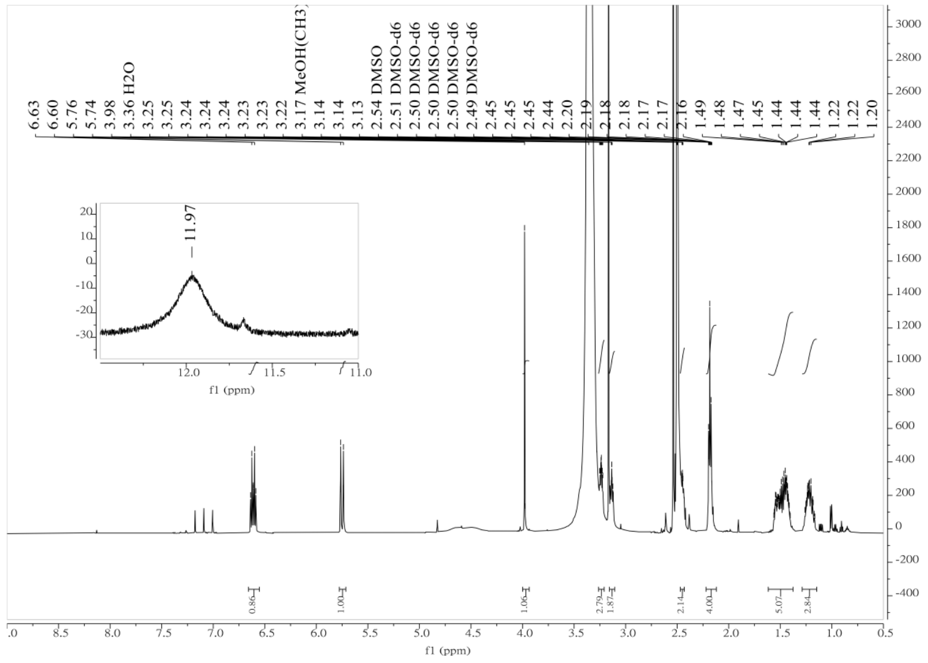

Supplementary Figure 6. ^{1}H NMR spectrum of collimonin C 1

COSY

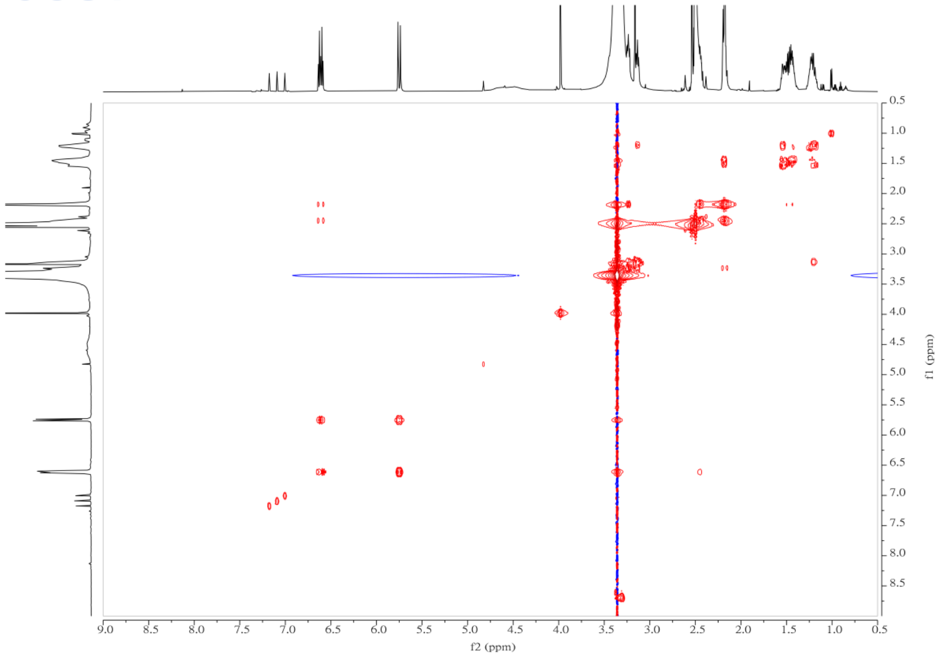

Supplementary Figure 7. ^{1}H-^{1}H COSY spectrum of collimonin C 1

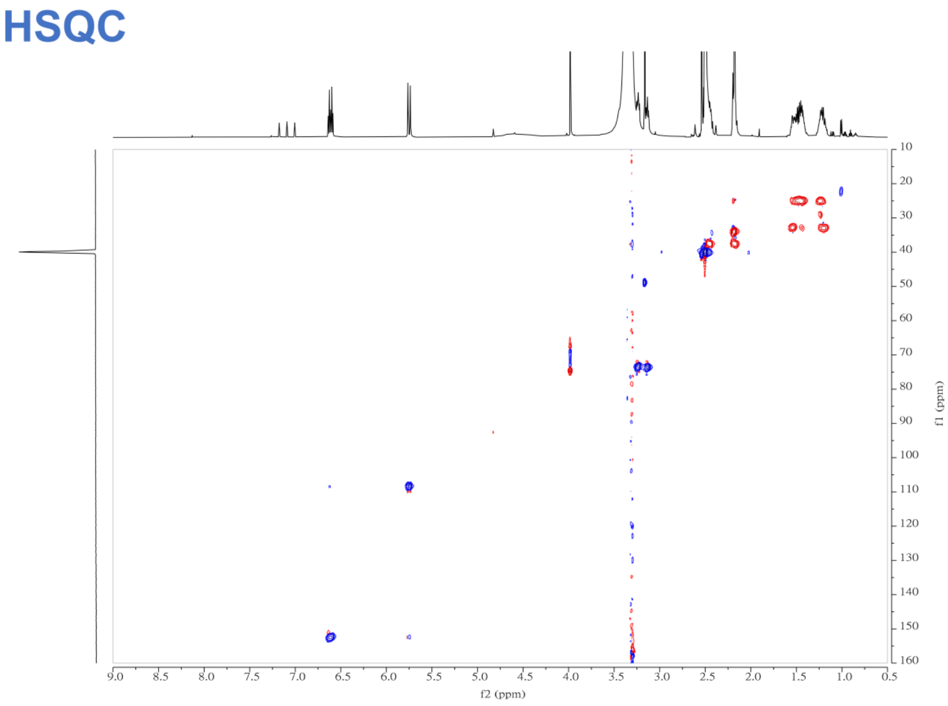

Supplementary Figure 8. HSQC spectrum of collimonin C 1

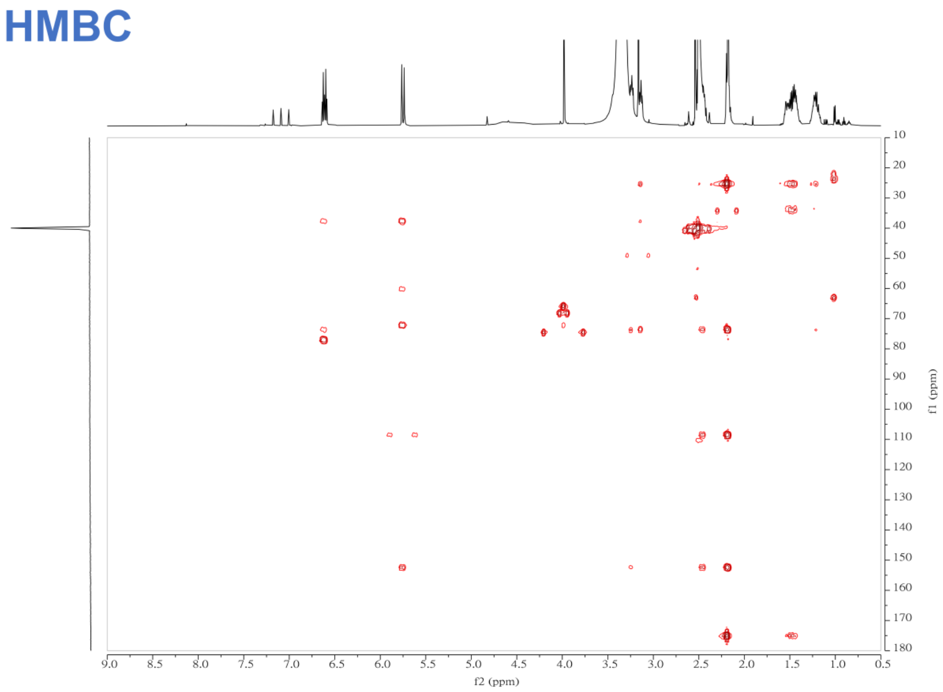

Supplementary Figure 9. HMBC spectrum of collimonin C 1

^{1}H NMR

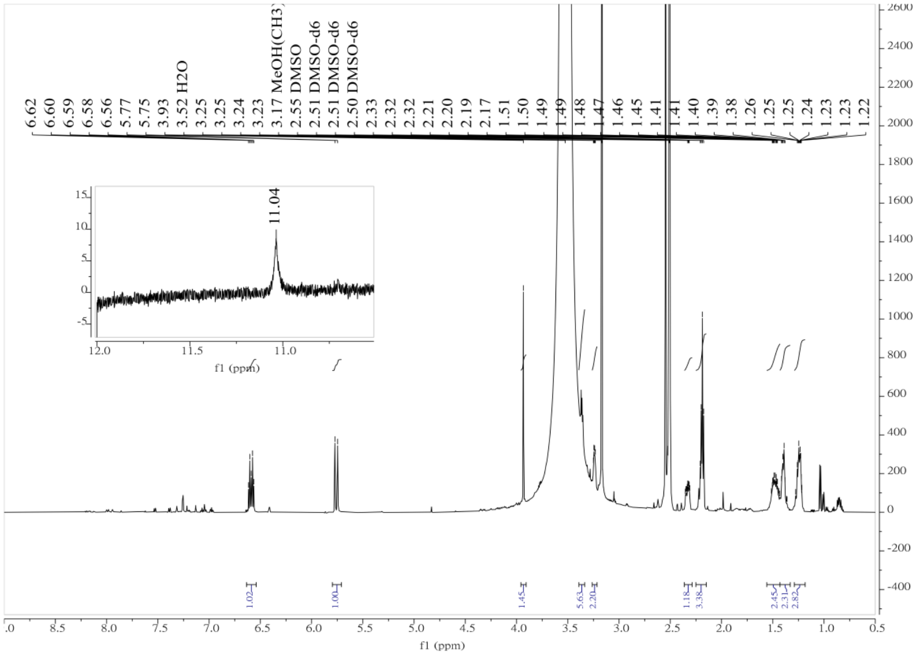

Supplementary Figure 10. ^{1}H NMR spectrum of collimonin D 2

COSY

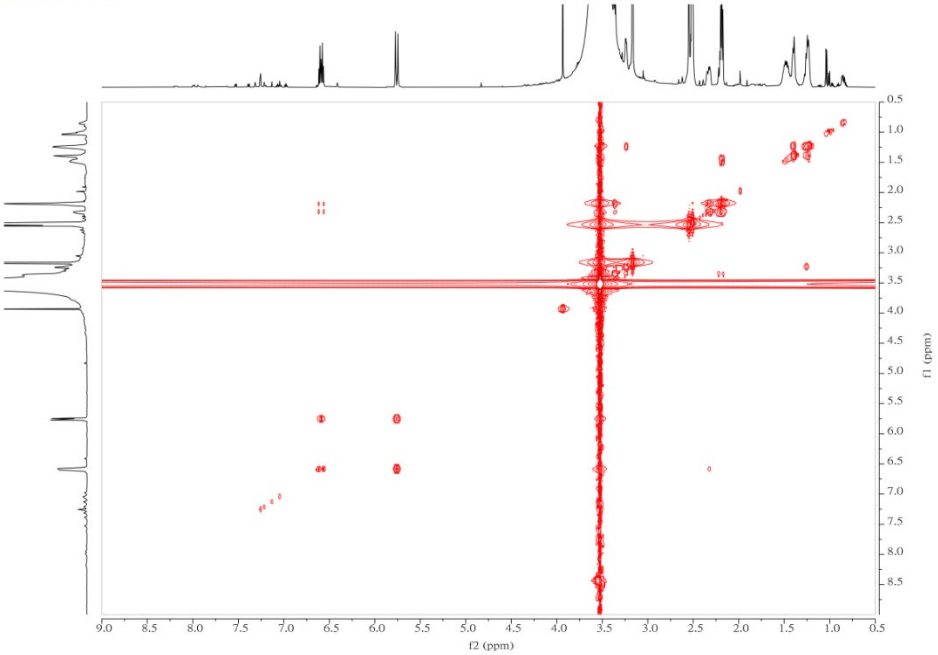

Supplementary Figure 11. ^{1}H-^{1}H COSY spectrum of collimonin D 2

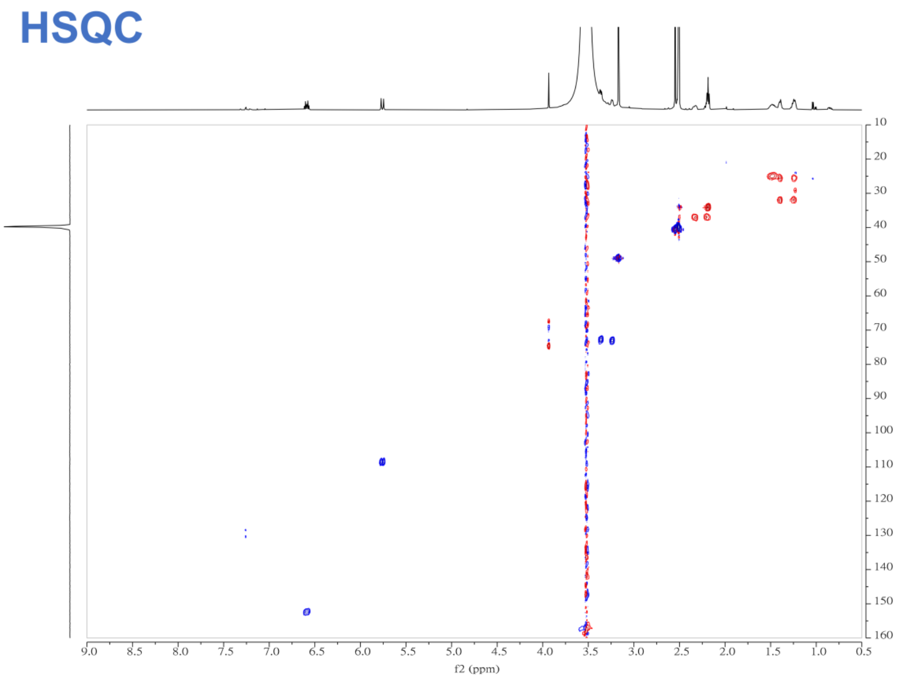

Supplementary Figure 12. HSQC spectrum of collimonin D 2

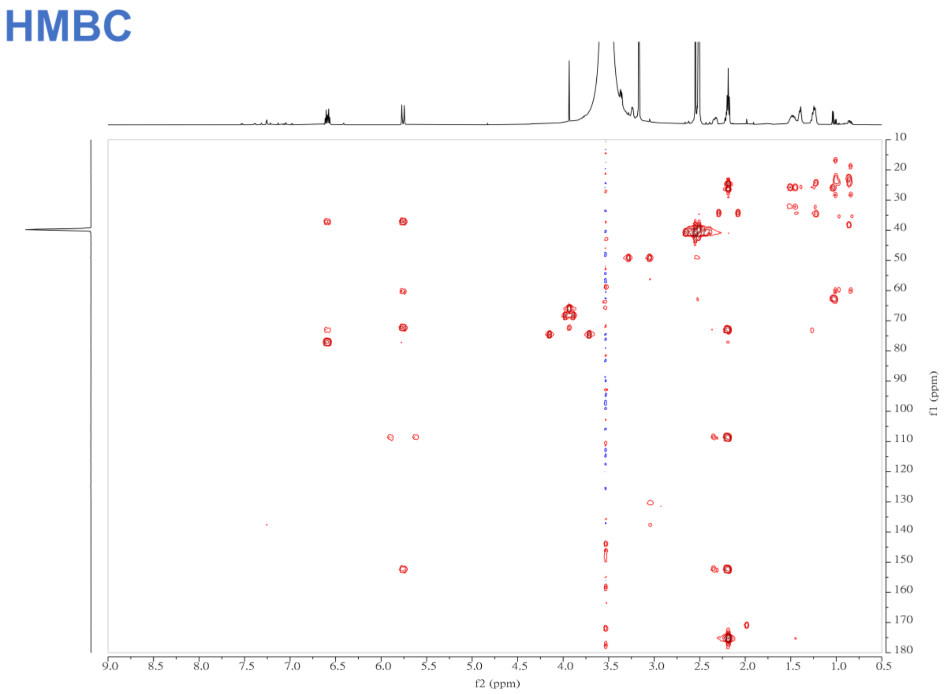

Supplementary Figure 13. HMBC spectrum of collimonin D 2

^{1}H NMR

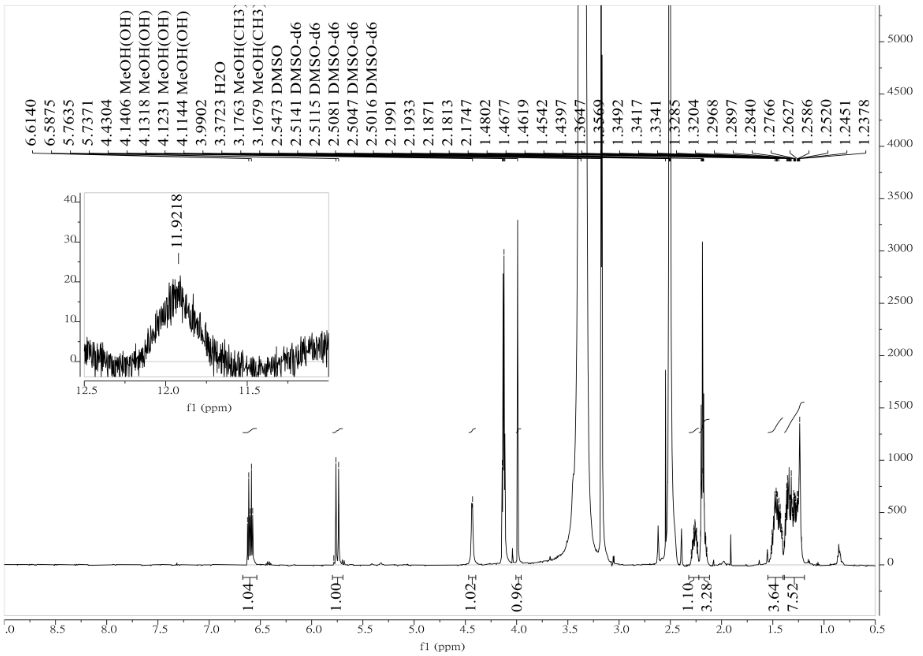

Supplementary Figure 14. ^{1}H NMR spectrum of massilin A 3

COSY

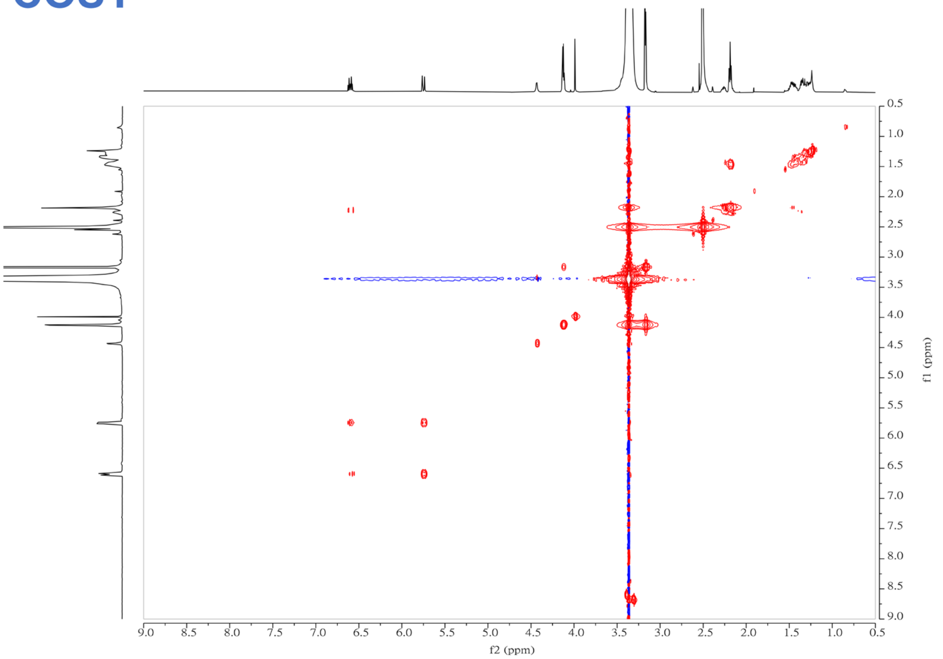

Supplementary Figure 15. ^{1}H-^{1}H COSY spectrum of massilin A 3

HSQC

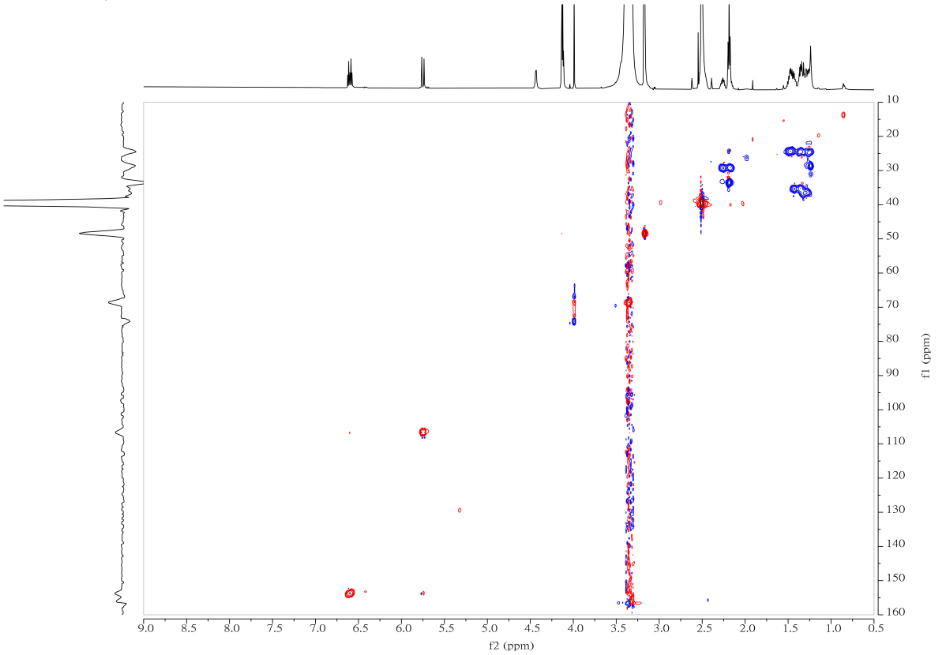

Supplementary Figure 16. HSQC spectrum of massilin A 3

HMBC

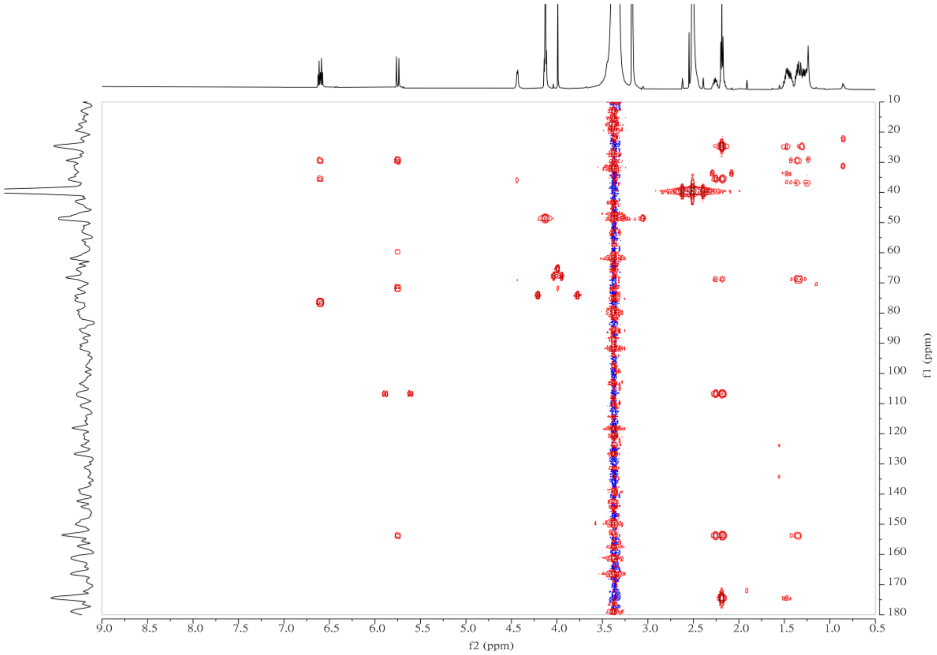

Supplementary Figure 17. HMBC spectrum of massilin A 3

^{1}H NMR

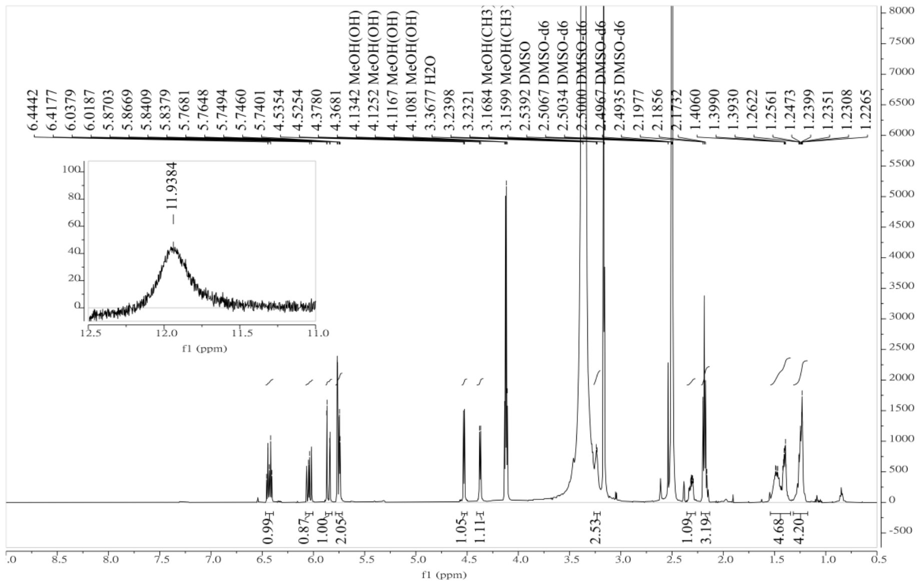

Supplementary Figure 18. ^{1}H NMR spectrum of massilin B 4

COSY

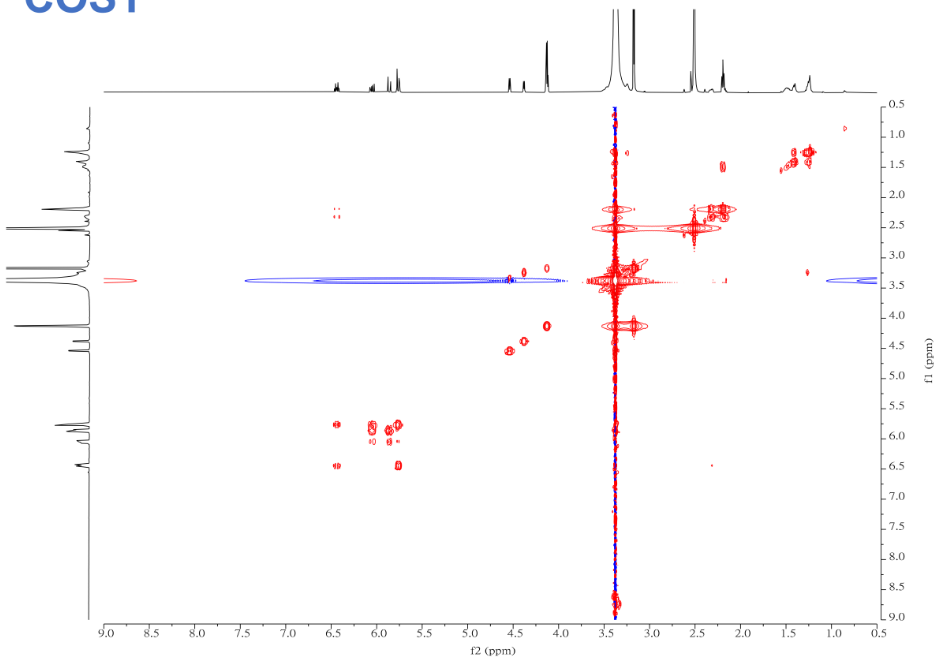

Supplementary Figure 19. ^{1}H-^{1}H COSY spectrum of massilin B 4

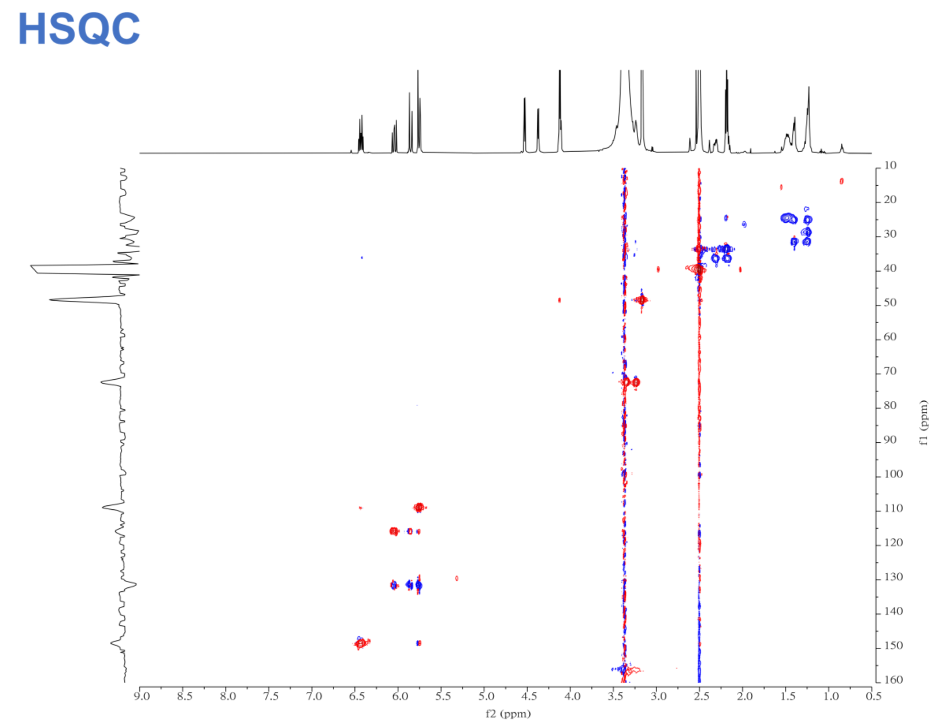

Supplementary Figure 20. HSQC spectrum of massilin B 4

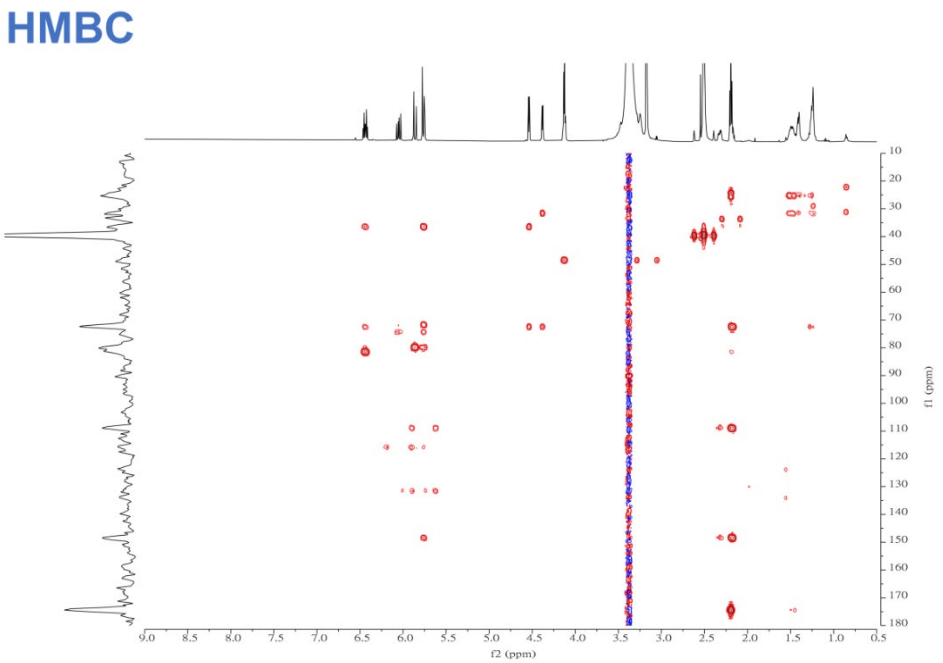

Supplementary Figure 21. HMBC spectrum of massilin B 4

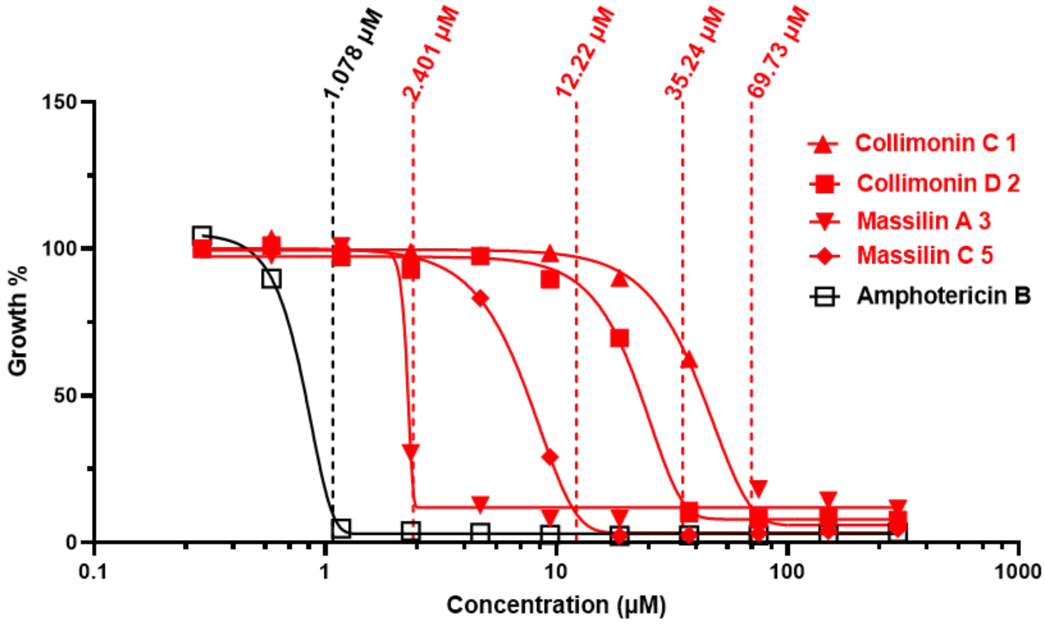

Supplementary Figure 22. Minimum inhibitory concentration (MIC) of polyynes and clinical drug against *C. albicans* ATCC18804 in YPD medium. Dose-response curves for cell viability treated by collimonin C **1** (red filled circle), collimonin D **2** (red filled square), massilin A **3** (red filled triangle), massilin C **5** (red filled diamond) and clinical drug amphotericin B (empty black square) in different concentrations (300.00, 150.00, 75.00, 37.50, 18.75, 9.38, 4.69, 2.34, 1.17, 0.59, 0.29 μM). The MIC value of each compound indicates above the dot-line. Each point was obtained from three biological replicates (*N*=3).

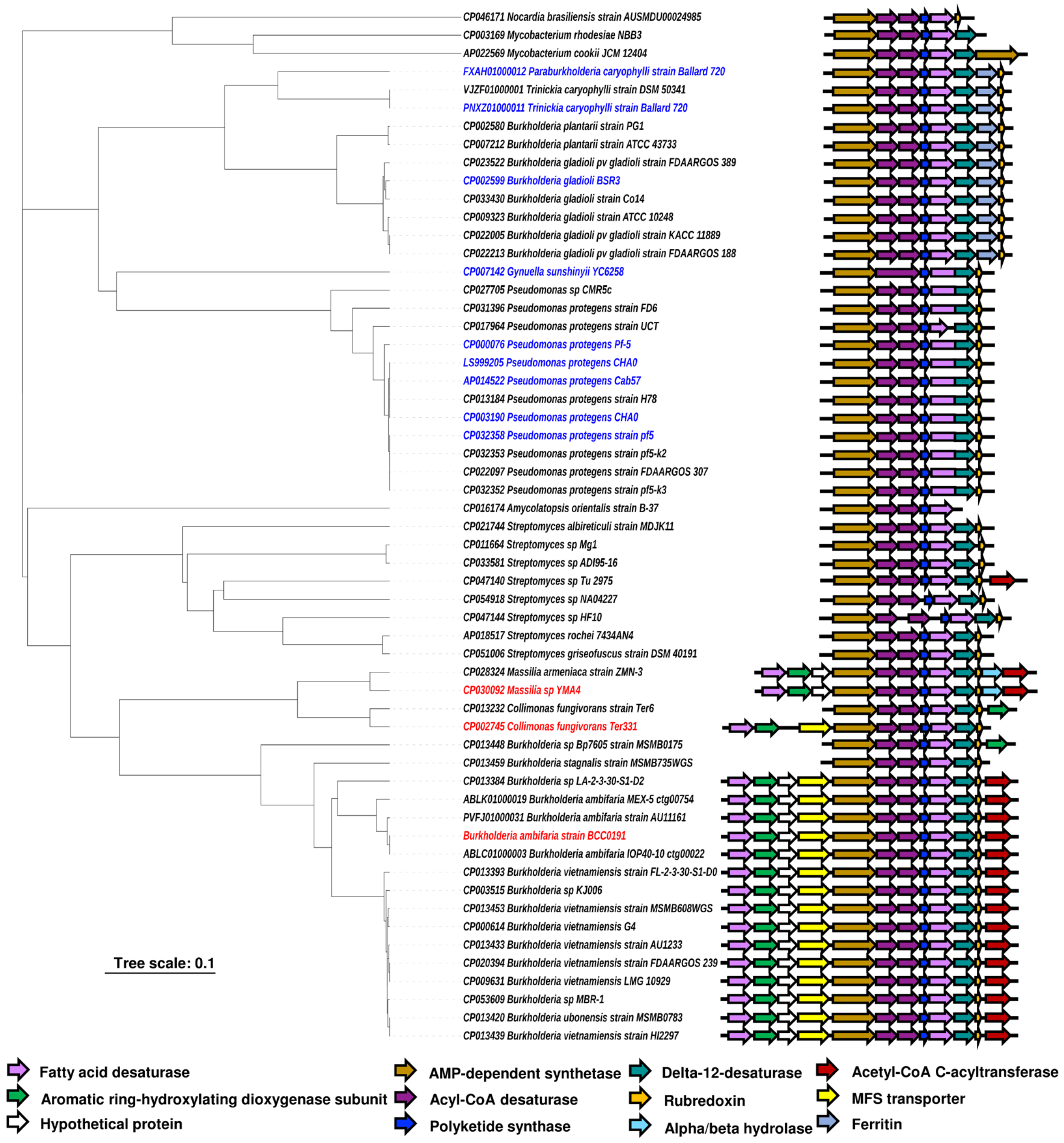

Supplementary Figure 23. Phylogenetic analysis of bacterial polyyne BGCs. The polyyne BGCs were mined from the NCBI BCT database (Version 2020) through protein sequence homology using polyyne BGC of *Massilia* sp. YMA4 (*masA* to *masL*) as a query by MultiGeneBlast. Strains colored in blue were reported to produce stearate-derived polyynes, and strains colored in red produced palmitate-derived polyynes. The bootstrapping phylogenetic tree was built with concatenated protein sequences of the gene cluster's conserved region (*masD* to *masI*) using MUSCLE alignment algorithm and distance estimated with 5000 bootstraps of UPGMA method in MEGA 10[1]. The tree scale indicated as 0.1 unit. The genes encoding functions were indicated in the legend.

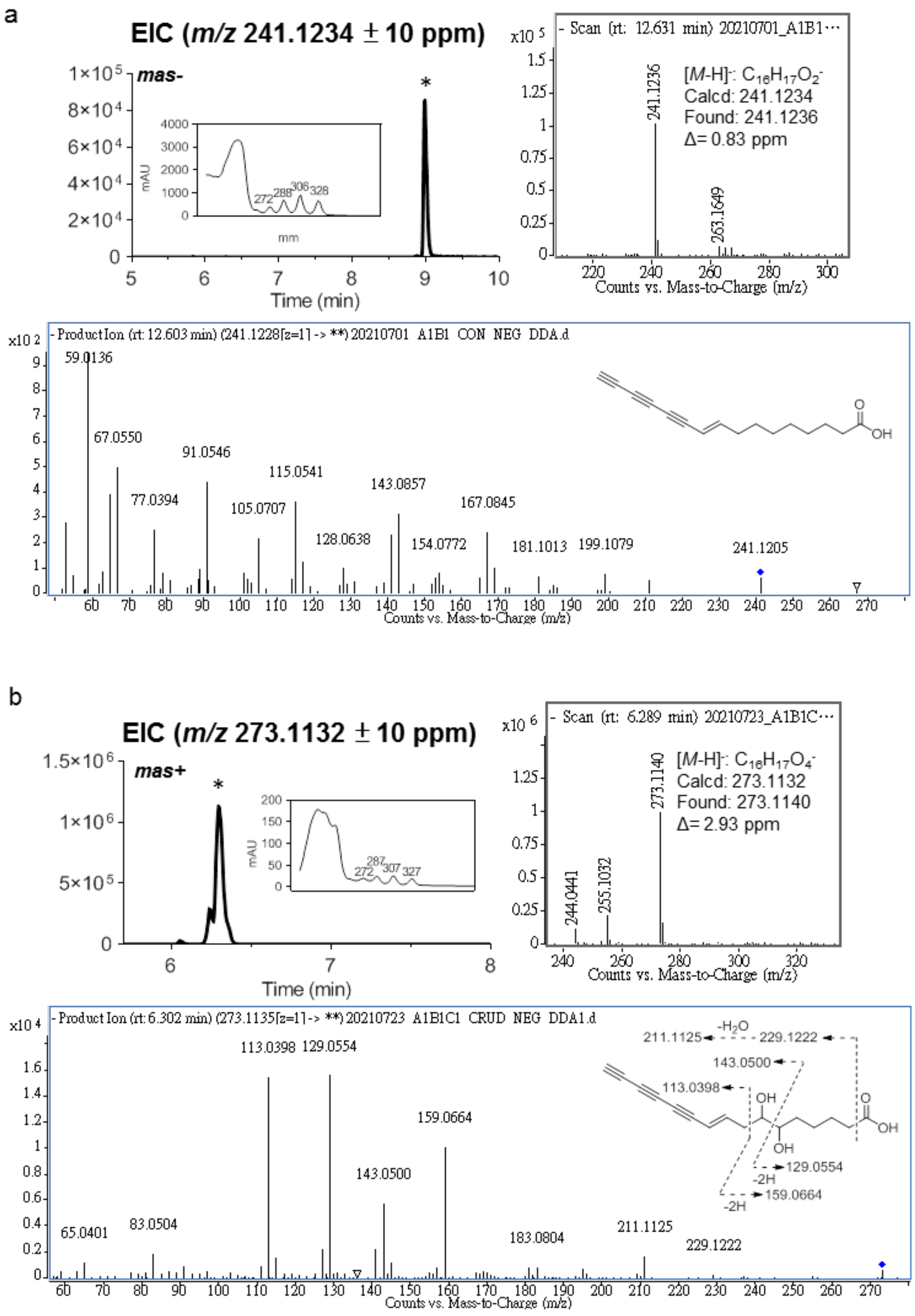

Supplementary Figure 24. LC-DAD-HRMS of the biosynthetic product from *E. coli* co-expression of *mas* genes. (**a**) Massilin C **5** produced by metabolic engineering strain *mas-.* (**b**) Collimonin C/D **1**, **2** produced by metabolic engineering strain *mas+*.

^{1}H NMR

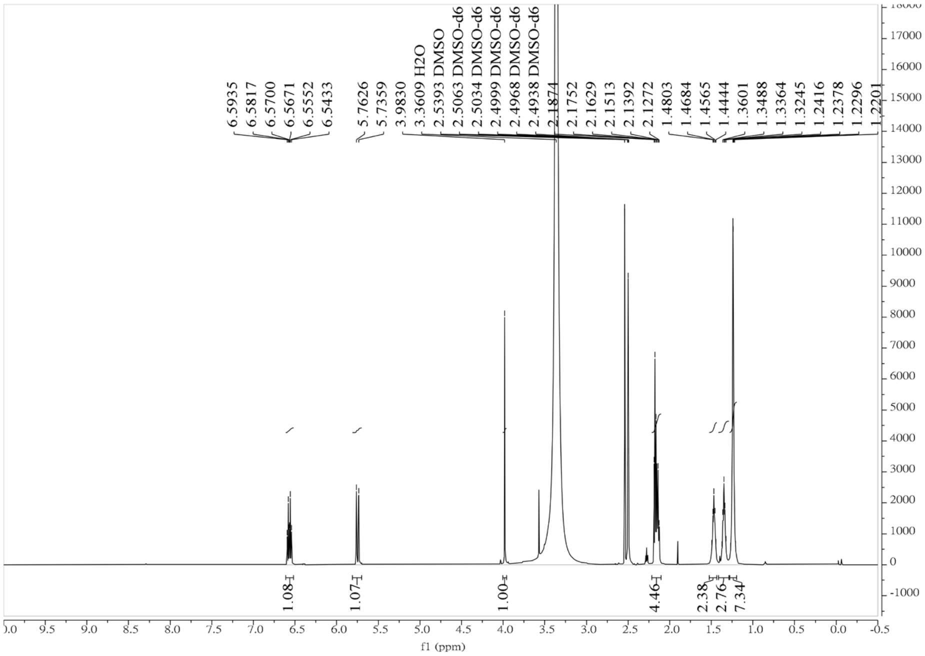

Supplementary Figure 25. ^{1}H NMR spectrum of massilin C 5

COSY

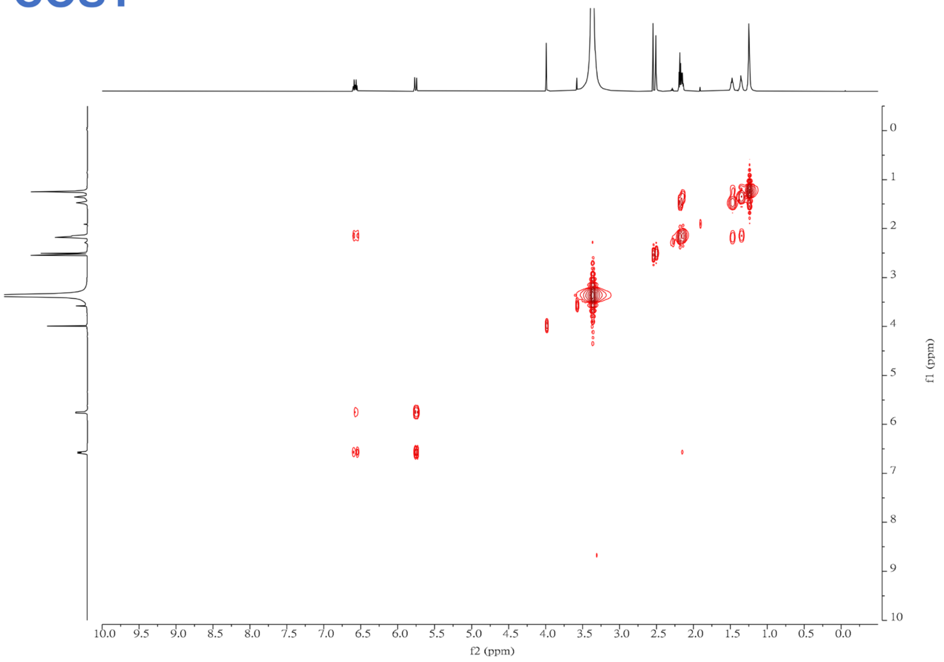

Supplementary Figure 26. ^{1}H-^{1}H COSY spectrum of massilin C 5

HSQC

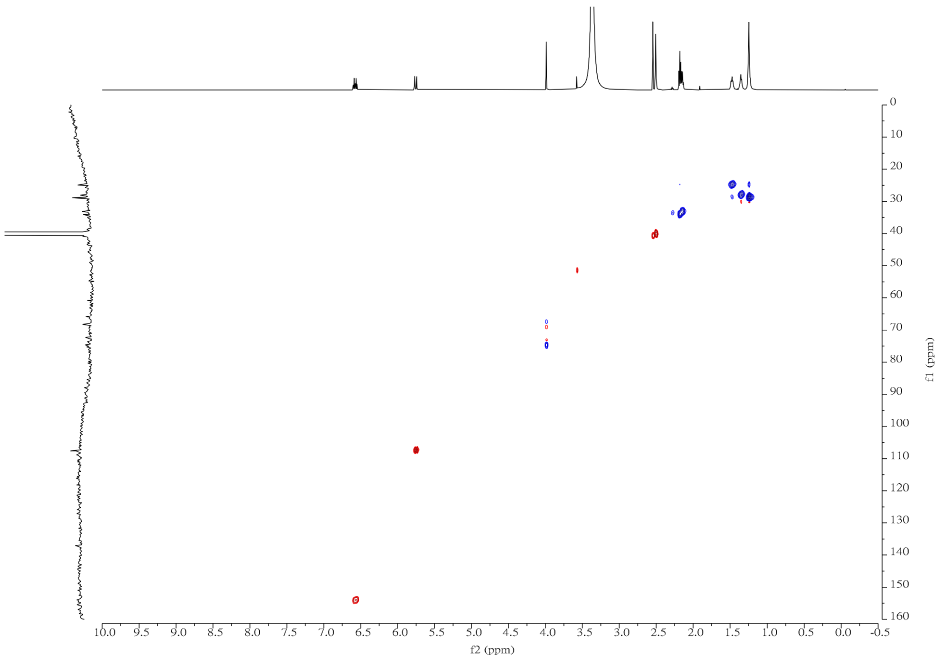

Supplementary Figure 27. HSQC spectrum of massilin C 5

HMBC

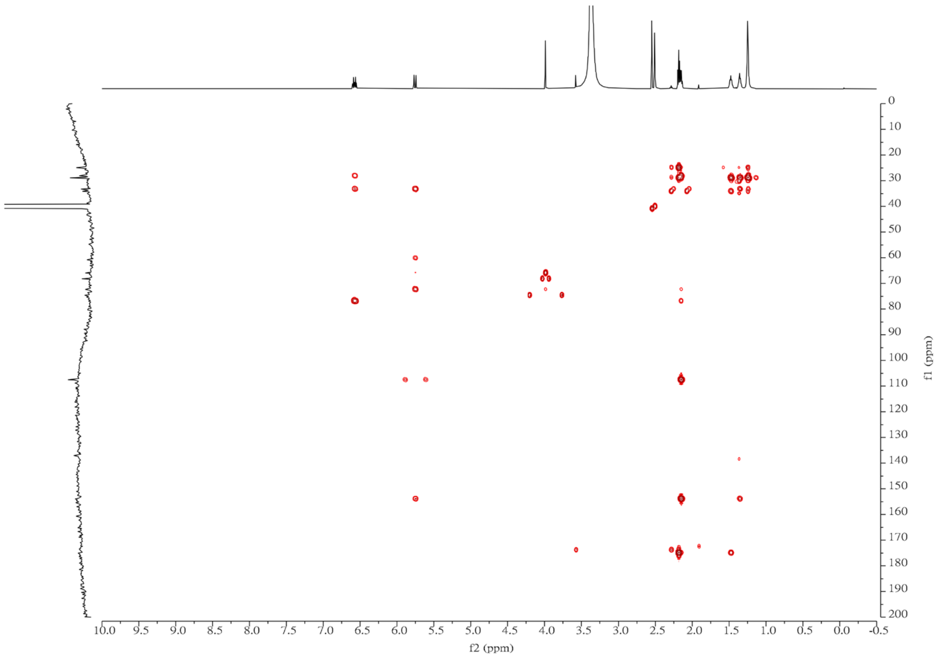

Supplementary Figure 28. HMBC spectrum of massilin C 5

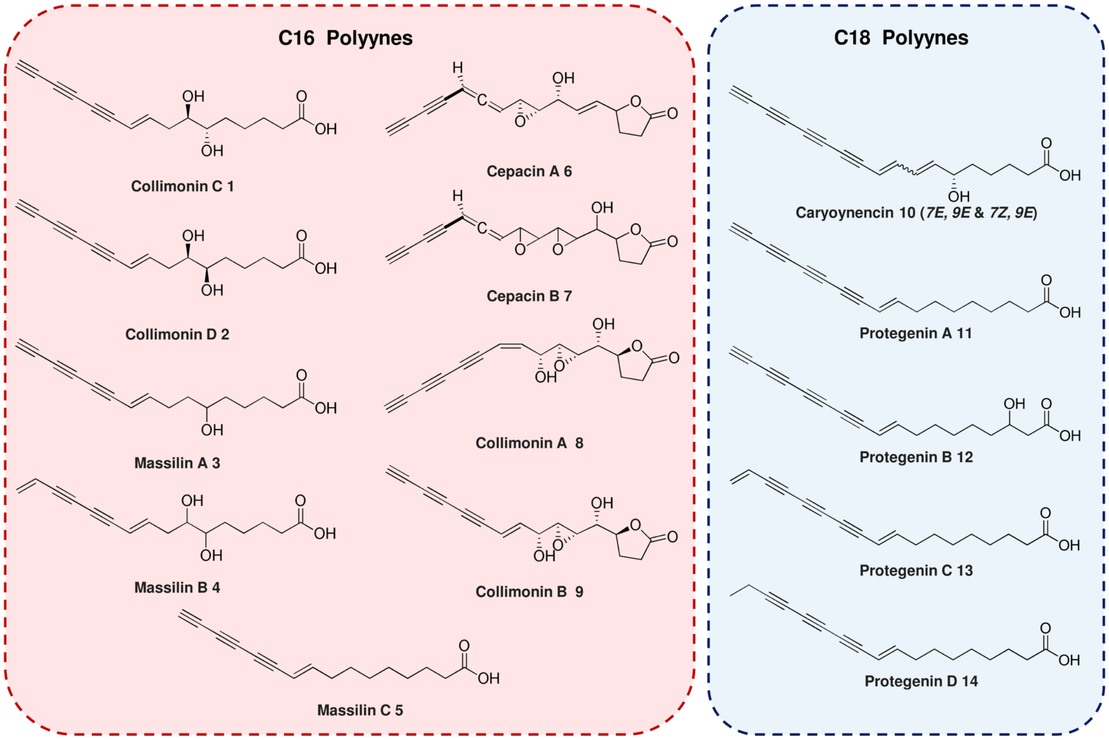

Supplementary Figure 29. The chemical structures of C16 and C18 polyynes annotated in Supplementary Figure 23. Colimonin C/D **1**, **2** and massilin A/B/C **3-5** were found in *Massilia* sp. YMA4 (in this study). Collimonin A/B/C/D **8**, **9**, **1**, **2** were found in *Collimonas fungivorans* Ter331[2]. Cepacin A/B **6**, **7** were found in *Burkholderia diffusa*[3] and *Burkholderia ambifaria* strains BCC0191[4]. Caryoynencin **10** was found in *Trinickia caryophylli* strain Ballard 720 (*Paraburkhilderia caryophylli*)[5] and *Burkholderia gladioli* BSR3[5]. Proteogenin A (Proteogecin) **11** was found in *Pseudomonas protegens* strains Cab57[6], Pf-5 and CHA0[7]. Proteogenin B/C/D **12-14** was found in *Pseudomonas protegens* strains Cab57[6].

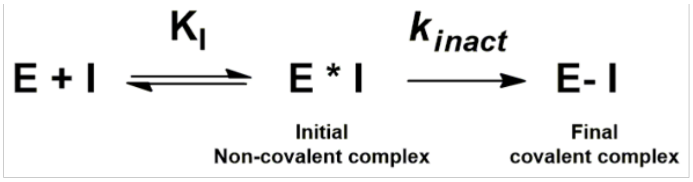

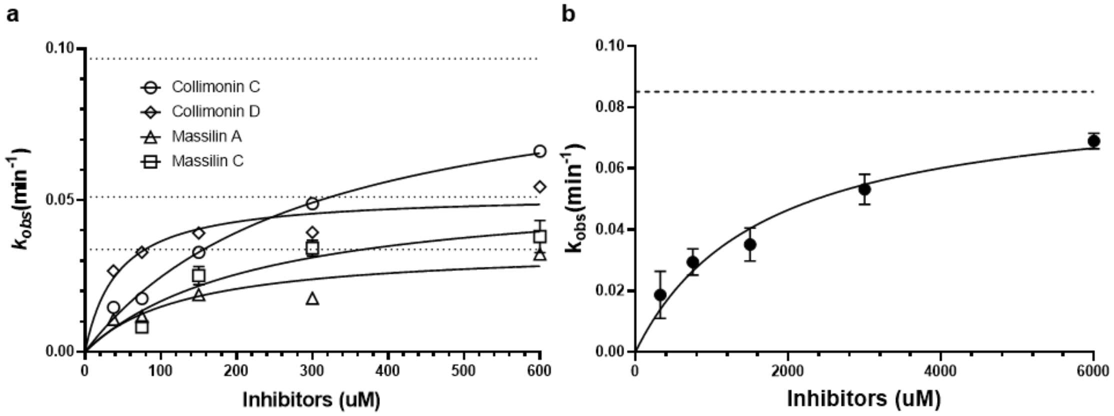

One site – Specific binding	Collimonin C	Collimonin D	Massilin A	Massilin C	Arecoline hydrobromide
**Best-fit values**					
K_{inact} (min^{-1})	0.09798	0.05208	0.03449	0.05477	0.08510
K_I (uM)	297.10	42.84	132.10	228.0	1663.00
**95% CI (profile likelihood)**					
K_{inact} (min^{-1})	0.06041 to 0.2239	0.03360 to 0.08768	0.02291 to 0.06074	0.03968 to 0.08535	0.07471 to 0.09847
K_I (uM)	103.7 to 1122	-3.562 to 243.0	37.22 to 463.1	99.05 to 568.9	1165 to 2386
**Goodness of Fit**					
Degrees of Freedom	38	43	37	20	28
R squared	0.4723	0.06669	0.3073	0.6411	0.8943
Sum of Squares	0.01299	0.04369	0.003650	0.001532	0.001087
Sy.x	0.01849	0.03188	0.009932	0.008754	0.006229

CI: Confidence interval.

Supplementary Figure 30. Kinetic evaluation of irreversible inhibitors and polyynes-MasL experiment detail. The covalent inhibitors described in this paper follow a two-step binding. Initial non-covalent binding to the enzyme under rapid equilibrium conditions was followed by slower covalent bond formation (reaction scheme). The progress curves for k_{inact} / K_I relationship between inhibitor concentration were recorded with CPM-labeling for coenzyme A production as acetyl-CoA acetyltransferase's residual activity. The inhibition reaction started from prior incubation of 10 μM MasL with various concentrations of polyynes (37.5, 75, 150, 300, 600 μM; panel a) or Arecoline hydrobromide, as a positive control for acetyl-CoA acetyltransferase inhibitor, (0.375, 0.75, 1.5, 3, 6 mM; panel b) for 15 and 30 min ($t_{1/2}^{\infty}$ of 15 and 30 min), followed by enzyme reaction and CPM-labeling for recorded the residual activity to calculate total occupancy. The progress curves used the hyperbolic regression model (Version 8, GraphPad Software, USA) to calculate the kinetic inhibition parameters. Each reaction point (time and concentration) was recorded at least three replicates for model building.

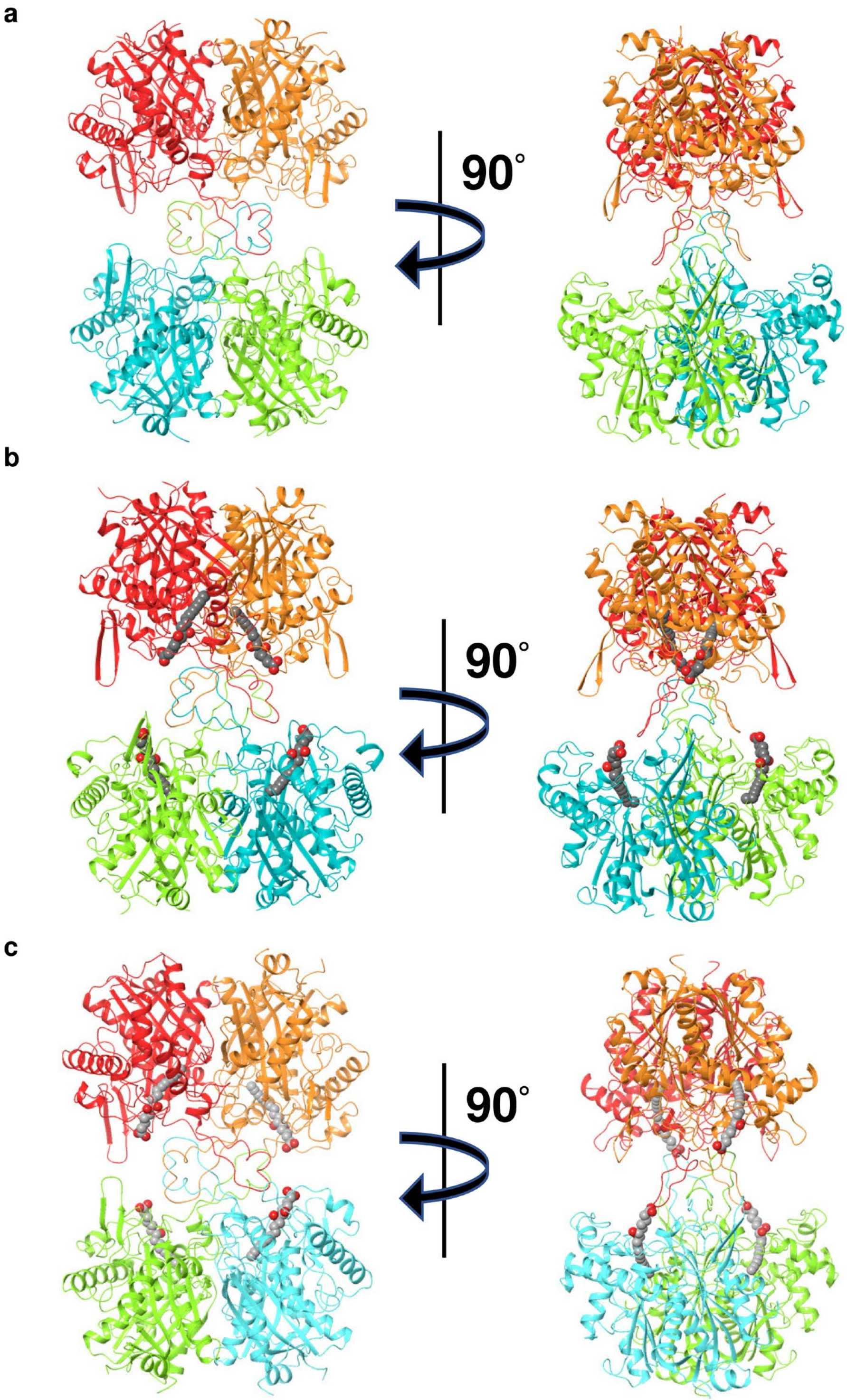

Supplementary Figure 31. Overall structures of MasL, MasL-collimonin C, and MasL-collimonin D complex. Tetrameric structures are shown as ribbon style for MasL (**a**), MasL-collimonin C (**b**), and MasL-collimonin D (**c**) complex. Four subunits of homogeneous tetramer are distinguished as red, orange, light green, and cyan. In addition, Collimonin C/D **1**, **2** is presented as space-filling style in the complex.

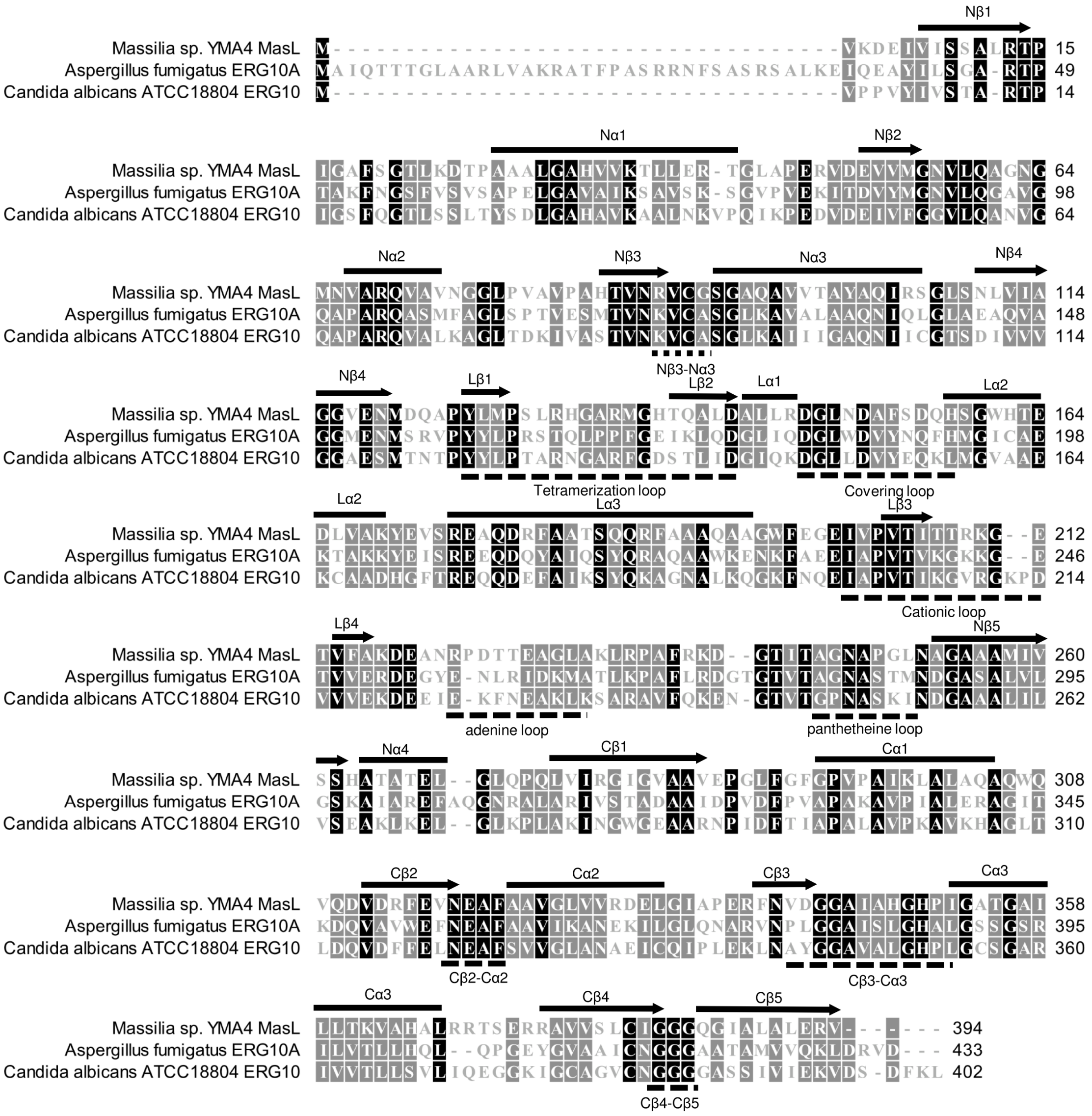

Supplementary Figure 32. Sequence alignment of acetyl-CoA acetyltransferases from different organisms. Amino acid sequence alignment of selected Acetyl-CoA acetyltransferases (*Massilia* sp. YMA4 MasL; *Aspergillus fumigatus* ERG10A (PDB ID **6L2C**[8]); *C. albicans* ATCC 18804 ERG10). Conserved residues over 80% were colored in gray, and 100% were colored in black.

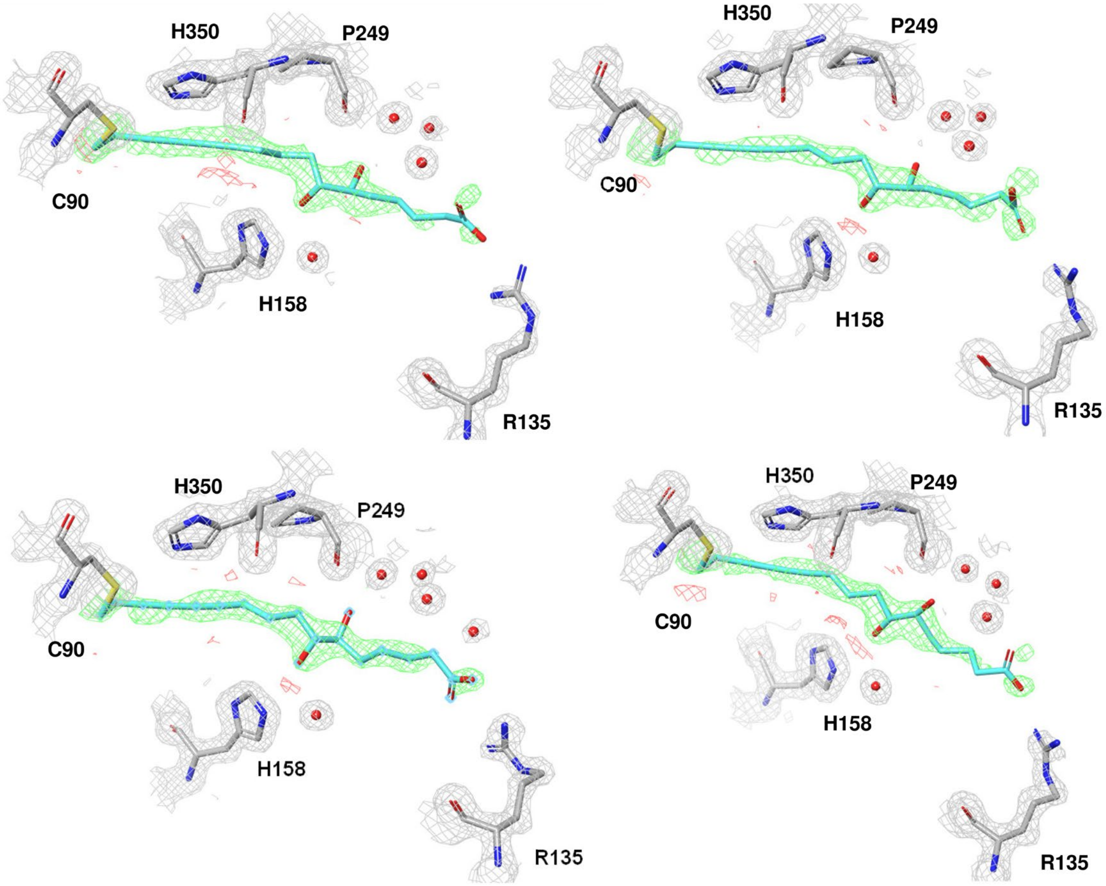

Supplementary Figure 33. Electron density map of collimonin C 1 in MasL reactive pocket. The initial $F_o - F_c$ electron density map contoured at 1.2σ around the collimonin C **1** (density in cyan) with refined $2F_o - F_c$ electron density contoured at 1.6σ for enzyme residues of MasL-collimonin C complex in four subunits. The carbon skeleton of collimonin C **1** is shown in cyan, and the carbons of catalytic residues are presented in grey, oxygens in red, nitrogens in blue, and sulfurs in yellow.

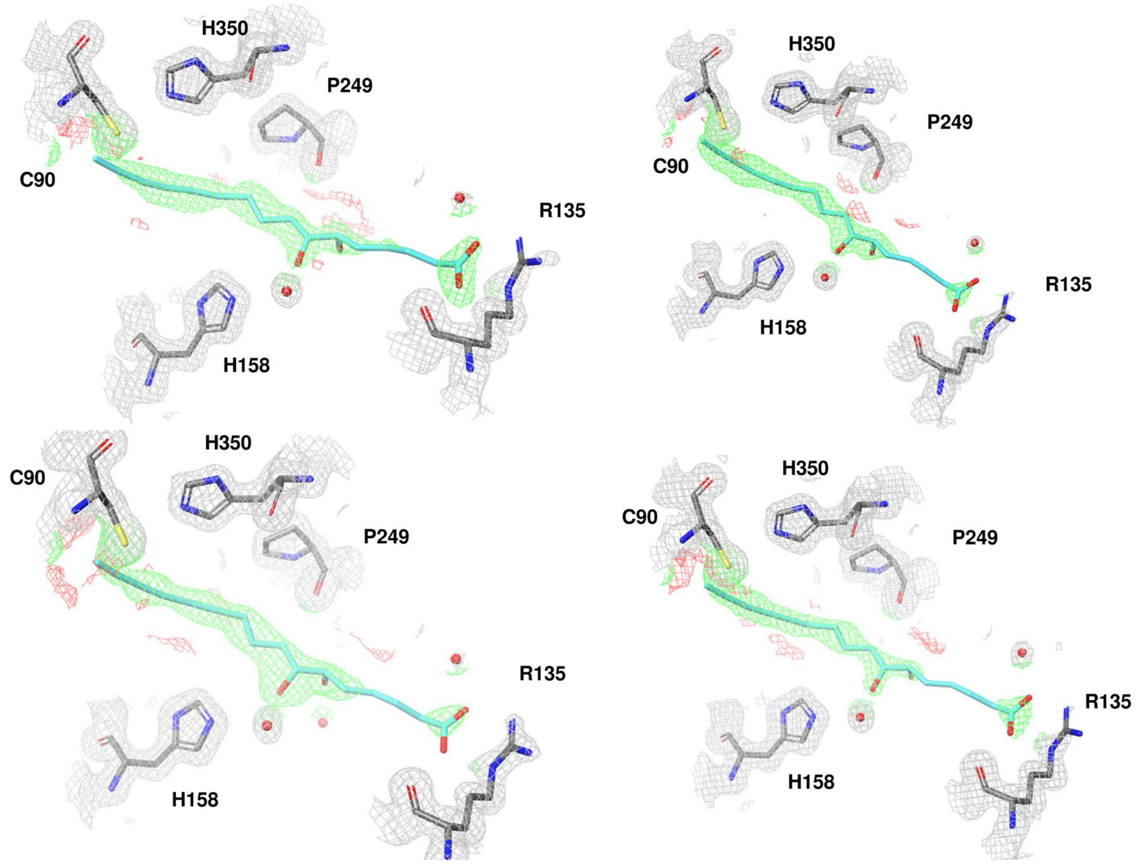

Supplementary Figure 34. Electron density map of collimonin D 2 in MasL reactive pocket. The initial $F_o - F_c$ electron density map contoured at 1.25σ around the collimonin D **2** (density in cyan) with refined $2F_o - F_c$ electron density contoured at 1.45σ for enzyme residues of MasL-collimonin D complex in four subunits. The carbon skeleton of collimonin D **2** carbons is shown in cyan. The carbons of catalytic residues are presented in grey, oxygens in red, nitrogens in blue, and sulfurs in yellow.

a

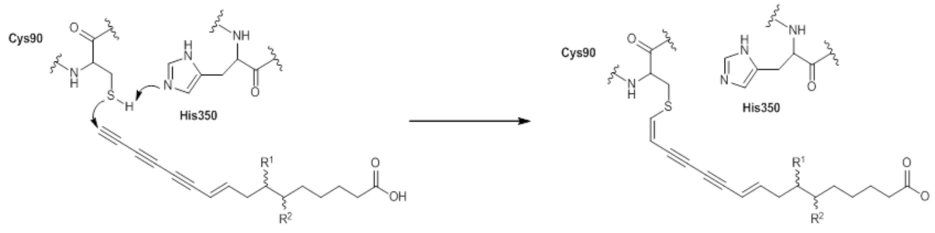

Collimonin C **1** R^1=OH, R^2=OH; (*6S*,*7R*)
Collimonin D **2** R^1=OH, R^2=OH; (*6R*,*7R*)
Massilin A **3** R^1=H, R^2=OH

b

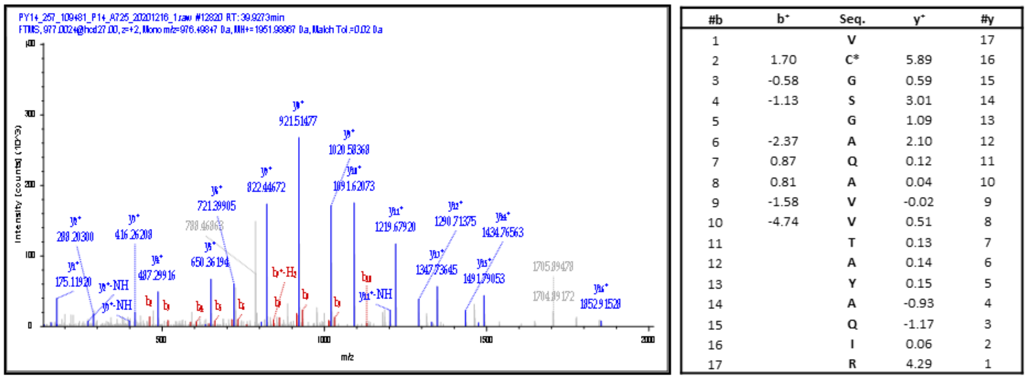

#b	b^+	Seq.	y^+	#y
1		V		17
2	1.70	C*	5.89	16
3	-0.58	G	0.59	15
4	-1.13	S	3.01	14
5		G	1.09	13
6	-2.37	A	2.10	12
7	0.87	Q	0.12	11
8	0.81	A	0.04	10
9	-1.58	V	-0.02	9
10	-4.74	V	0.51	8
11		T	0.13	7
12		A	0.14	6
13		Y	0.15	5
14		A	-0.93	4
15		Q	-1.17	3
16		I	0.06	2
17		R	4.29	1

c

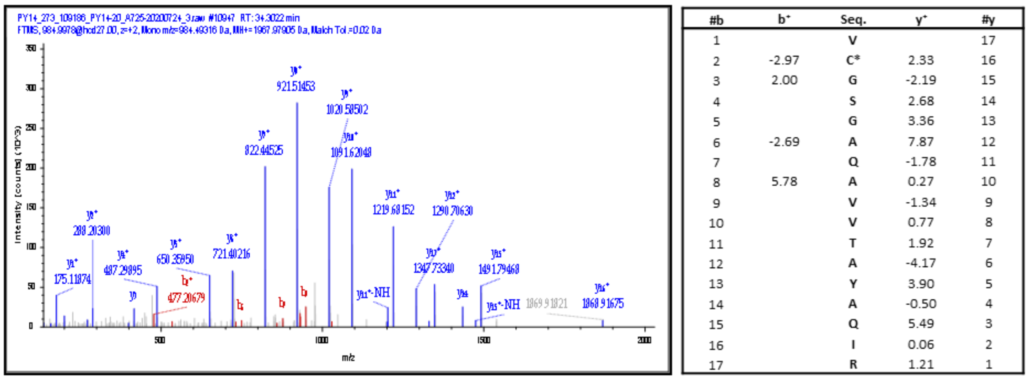

#b	b^+	Seq.	y^+	#y
1		V		17
2	-2.97	C*	2.33	16
3	2.00	G	-2.19	15
4		S	2.68	14
5		G	3.36	13
6	-2.69	A	7.87	12
7		Q	-1.78	11
8	5.78	A	0.27	10
9		V	-1.34	9
10		V	0.77	8
11		T	1.92	7
12		A	-4.17	6
13		Y	3.90	5
14		A	-0.50	4
15		Q	5.49	3
16		I	0.06	2
17		R	1.21	1

Supplementary Figure 35. Bottom-up proteomics analysis of MasL treated by polyynes. (**a**) A scheme illustrated that polyynes (with terminal alkyne) are electrophiles for thiol−alkyne addition targeting MasL reactive cysteine residue Cys90. NanoLC-Q-HCD-orbitrap tandem mass spectra and annotation of massilin A **3** (**b**) and collimonin C/D **1**, **2** (**c**) derived covalent modification of trypsin-digested MasL peptides. The annotated ion peaks are colored in blue (y ion) and red (b ion). Mass errors are shown in ppm in the annotation table. Asterisks in the sequence indicate the modified cysteine residue. Other modification peptides were listed and provided in **Supplementary Data 3**.

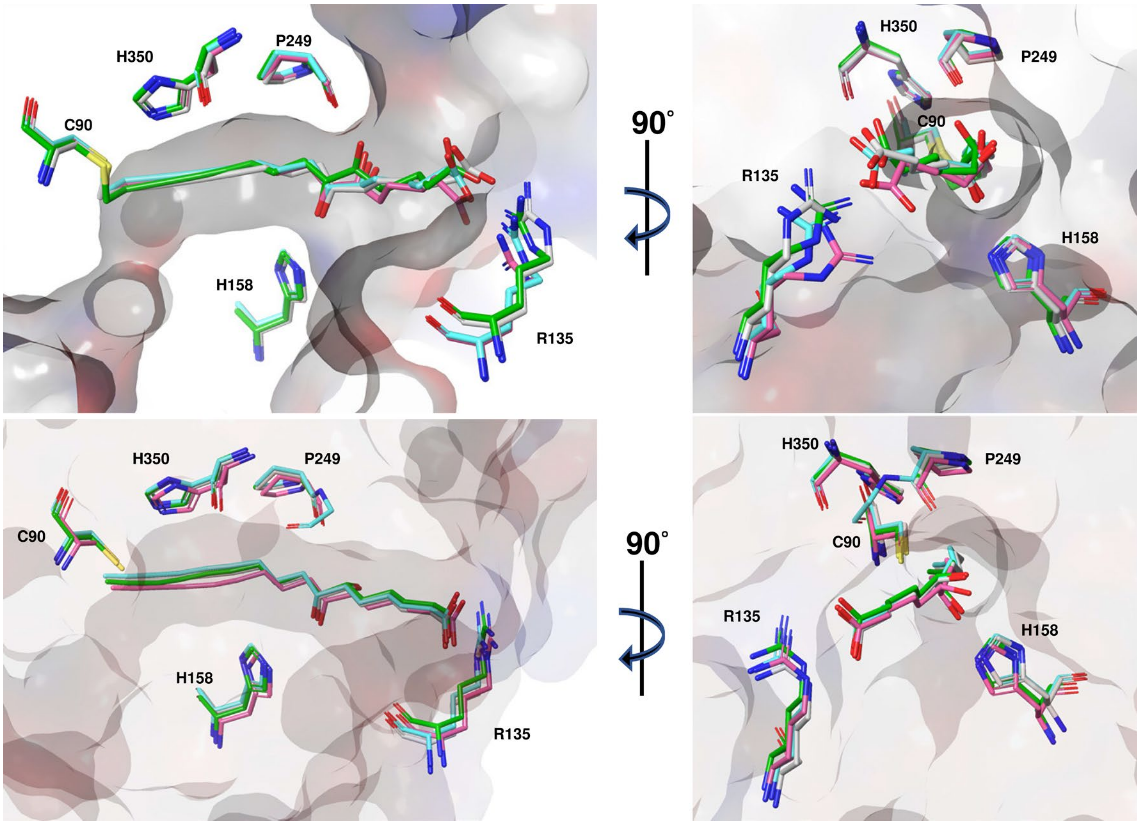

Supplementary Figure 36. Magnification view of MasL covalently modified by collimonin C/D 1, 2. Superimposition of collimonin C **1** in four subunits (top) and collimonin D **2** in four subunits (bottom), colored gray, cyan, green, and pink for each chain, respectively. The residues involved in hydrogen interactions are represented in stick representation with their respective sequence identities. The protein surface of MasL subunit A is colored with the corresponding residual charge for positive (blue) and negative (red).

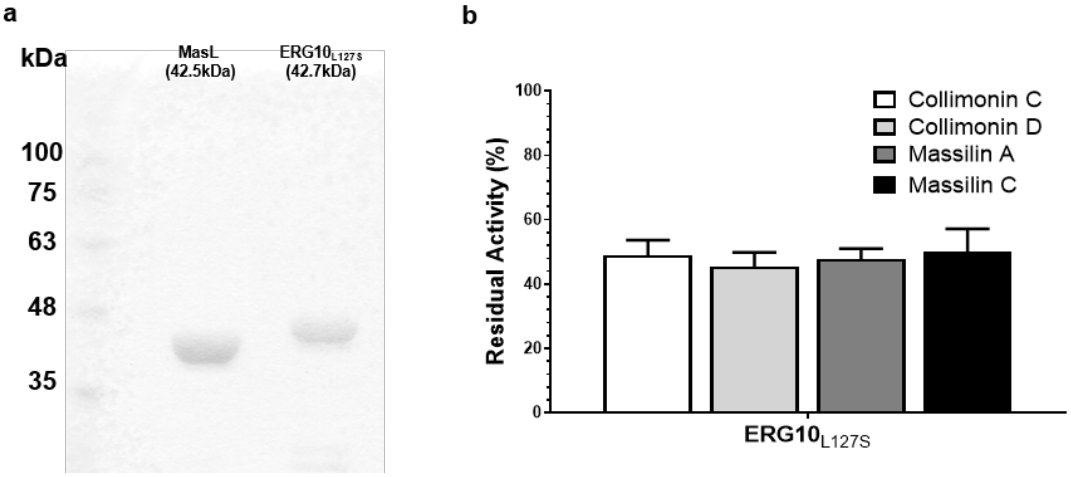

Supplementary Figure 37. Residual enzyme activity of *C. albicans* ERG10 treated by polyynes. (**a**) SDS-PAGE of recombinant acetyl-CoA acetyltransferases (MasL from *Massilia* sp. YMA4; $ERG10_{L127S}$ from *C. albicans* ATCC18804 (**b**) Residual enzyme activity of $ERG10_{L127S}$ treated by polyynes. Reaction mixtures contained 20 μM enzyme and 100 μM polyyne for 2 h reaction followed by 200 μM acetyl-CoA for 1 h substrate reaction. Residual activity was detected by the CPM labeling method and normalized with 2% DMSO as a control treatment. Each residual activity was obtained from three biological replicates (*N*=3-4). Error bar shows the standard error of the mean (SEM).

a

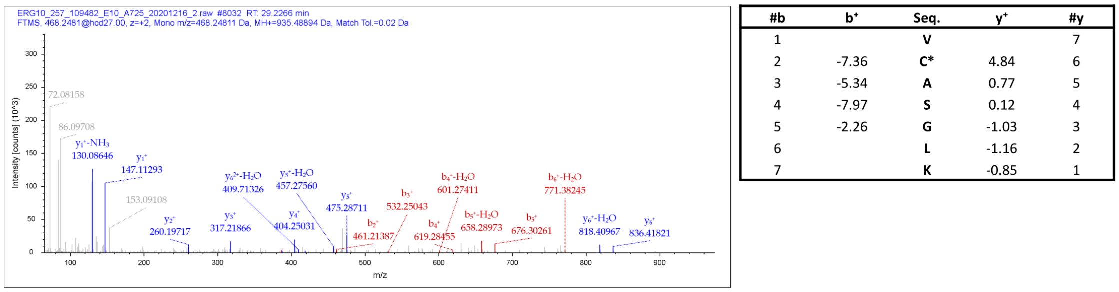

#b	b⁺	Seq.	y⁺	#y
1		V		7
2	-7.36	C*	4.84	6
3	-5.34	A	0.77	5
4	-7.97	S	0.12	4
5	-2.26	G	-1.03	3
6		L	-1.16	2
7		K	-0.85	1

b

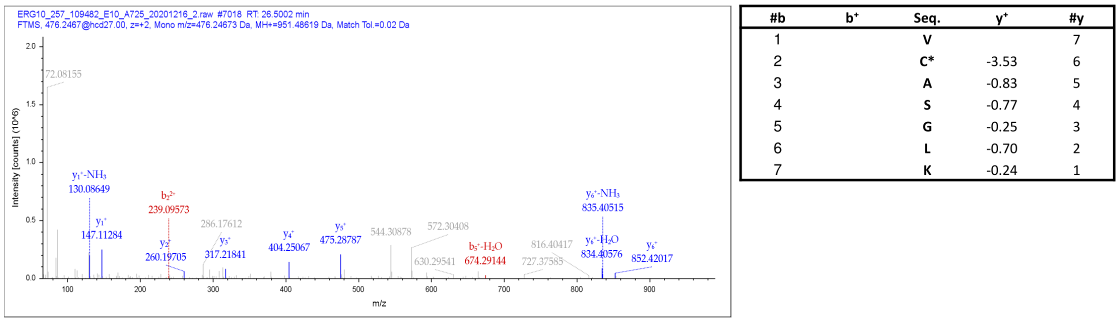

#b	b⁺	Seq.	y⁺	#y
1		V		7
2		C*	-3.53	6
3		A	-0.83	5
4		S	-0.77	4
5		G	-0.25	3
6		L	-0.70	2
7		K	-0.24	1

Supplementary Figure 38. Bottom-up proteomics analysis of *C. albicans* ERG10 treated by polyynes. NanoLC-Q-HCD-orbitrap tandem mass spectra and annotation of massilin A **3** (**a**) and collimonin C/D **1**, **2** (**b**)-derived covalent modification trypsin-digested ERG10 peptides (Cys90) Mass errors are shown in ppm in the annotation table. Asterisks in the sequence indicate the modified cysteine residue. Other modification peptides were listed and provided in Data 3.

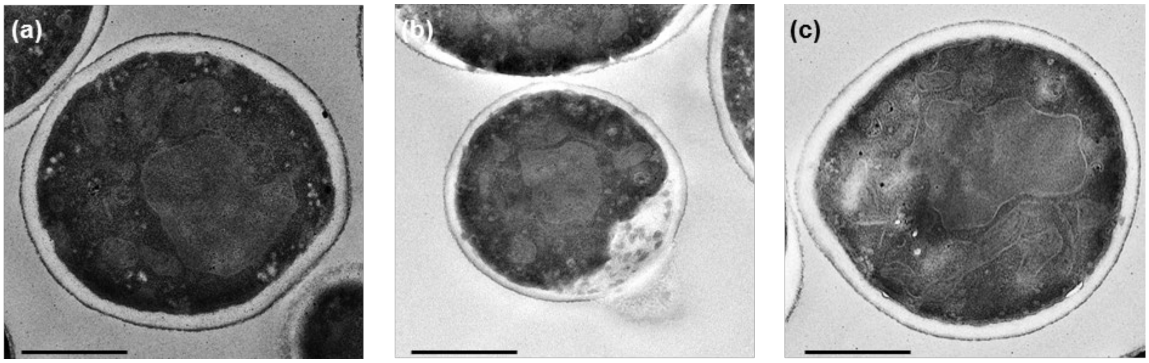

Supplementary Figure 39. Transmission electron microscopy (TEM) images of polyyne-treated *C. albicans* ATCC18804. *C. albicans* were respectively treated with Mock (**a**), 1 mg/mL ethyl acetate (EA) crude extract of WT (**b**), and 1 mg/mL EA crude extract of strain YMA4::*masH* (**c**). Scale bar indicates 1 μm distance.

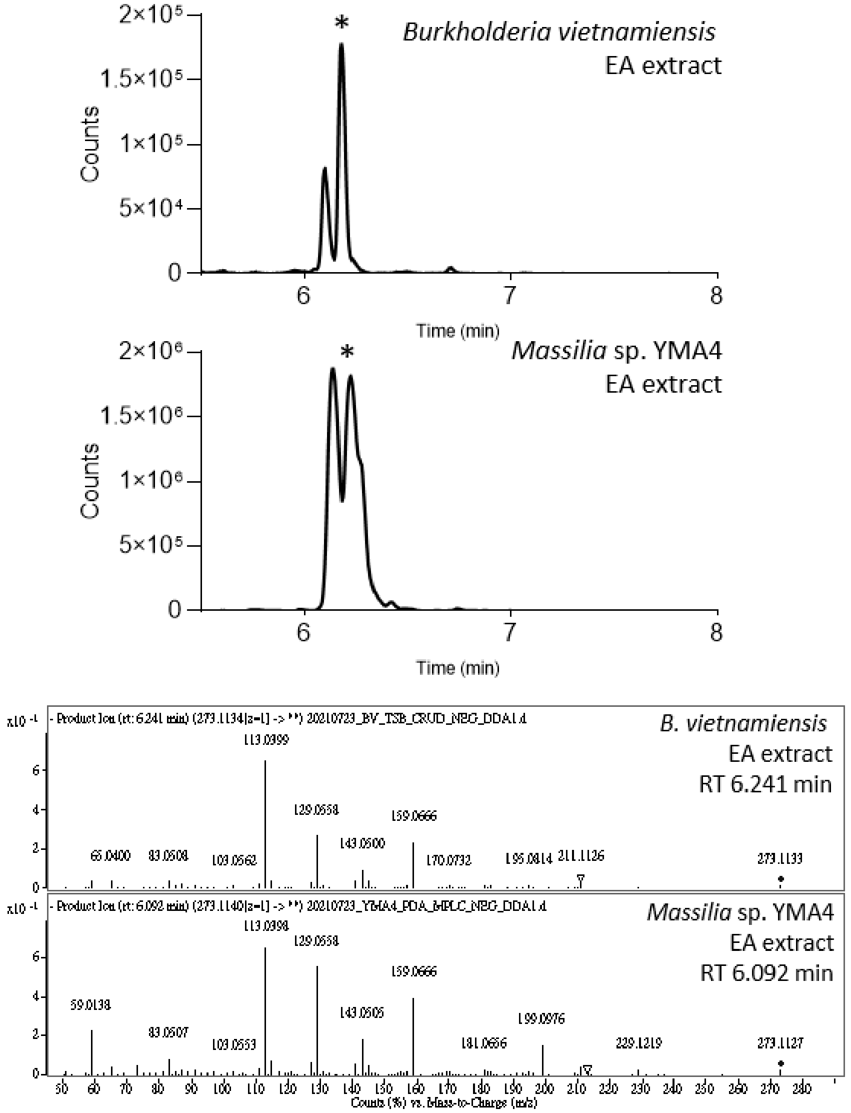

Supplementary Figure 40. LC-HRMS/MS profiles and tandem mass spectra of polyynes from *B. vietnamiensis* LMG 10929 and *Massilia* sp. YMA4. EIC (*m/z* 273.1132 ± 10 ppm) represented dihydroxyl polyyne features.

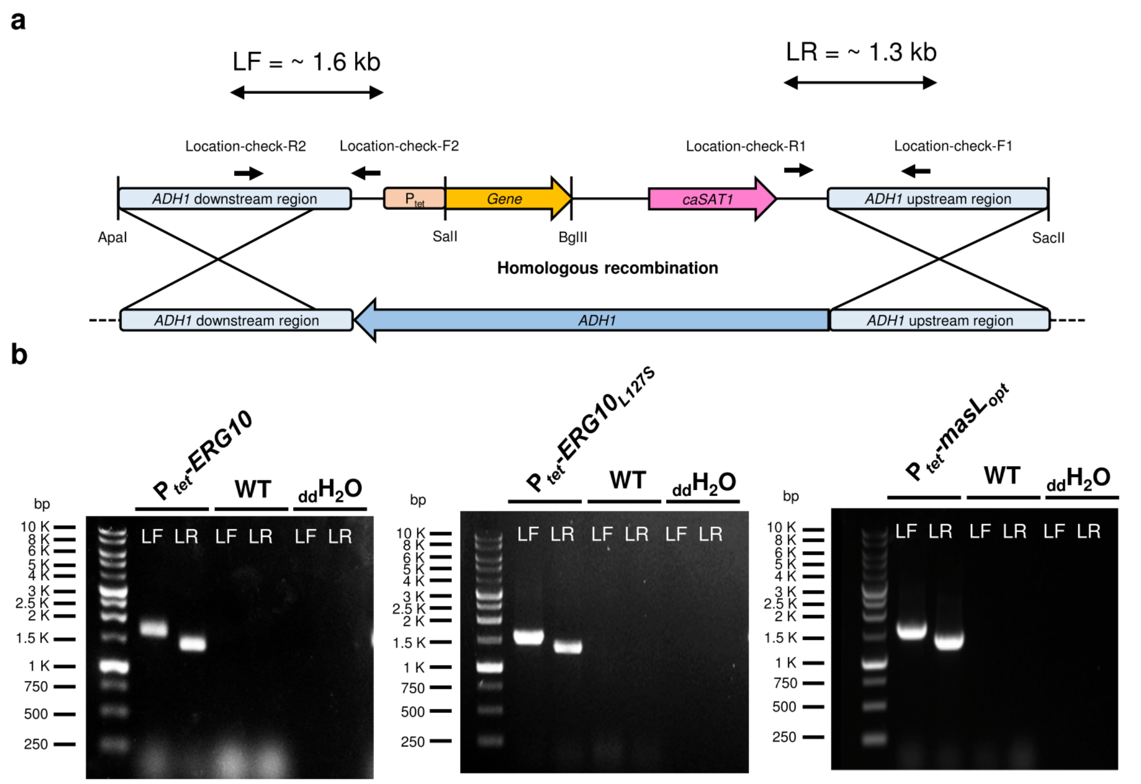

Supplementary Figure 41. Tetracycline-inducible expression system in *C. albicans* ATCC18804. (**a**) Scheme of the inducible overexpression of *CaERG10* and Codon optimized *masL* (*masL*$_{opt}$) constructed by homologous recombination replacing with *ADH1* gene in the chromosome. (**b**) PCR check results of tetracycline-inducible *ERG10*, *ERG10*$_{L127S}$, and *masL*$_{opt}$ strains. The positions of the primer sets are shown in (**a**).

Supplementary Tables

Supplementary Table 1. ^{1}H and ^{13}C NMR of collimonin C 1 in C_2D_6OS

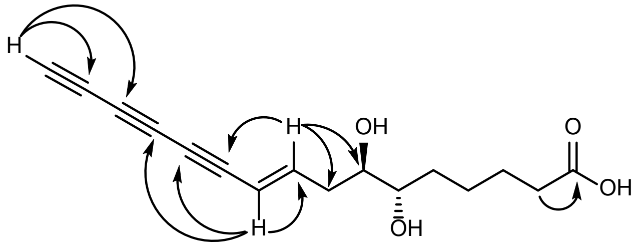

⟶ Key HMBC correlations of collimonin C **1**

No.	δ_H (J in Hz)		δ_C	
	this study	Kai K et al.[2]	this study	Kai K et al.[2]
1	11.97, br s, COOH	11.95, br s, COOH	174.4, COOH	175.0, COOH
2	2.18, t, $J_{2,3}$ 7.22	2.17, t, $J_{2,3}$ 7.2	34.0	34.3
3	1.47, m	1.46, m	25.0	25.2
4	1.21,1.53, m	1.21, 1.50, m	25.3	25.4
5	1.18,1.53, m	1.18, 1.52, m	32.9	33.1
6	3.13, m	3.13, m	73.6	73.8
7	3.24, m	3.23, m	73.4	73.6
8	2.17, m; 2.45, m	2.17, 2.44, m	37.5	37.8
9	6.61, dt, $J_{9,10}$ 16.0, $J_{9,8}$ 7.38	6.60, dt, $J_{9,10}$ 16.0, $J_{9,8}$ 7.4	152.1	152.4
10	5.75, d, $J_{10,9}$ 16.0	5.74, d, $J_{10,9}$ 16.0	107.9	108.6
11			77.0	77.1
12			60.2	60.2
13			72.2	72.2
14			65.9	65.9
15			68.1	68.2
16	3.98, s	3.97, s	74.2	74.6

*600 MHz for ^{1}H NMR
†^{13}C chemical shifts were assigned based on HSQC and HMBC

Supplementary Table 2. ^{1}H and ^{13}C NMR of collimonin D 2 in C_2D_6OS

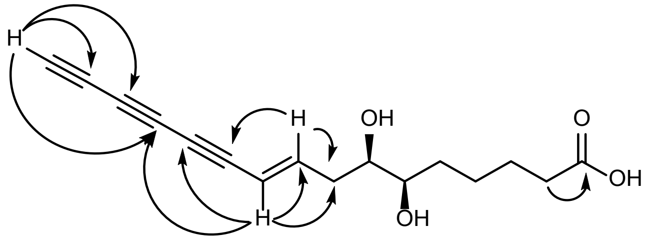

⟶ Key HMBC correlations of collimonin D **2**

No.	δ_H (*J* in Hz)		δ_C	
	this study	Kai K et al.[2]	this study	Kai K et al.[2]
1	11.04, br s, COOH	11.97, br s, COOH	175.0, COOH	174.4, COOH
2	2.18, t, $J_{2,3}$ 7.20	2.18, t, $J_{2,3}$ 7.2	34.0	33.7
3	1.47, m	1.50, m	25.0	24.6
4	1.24,1.40, m	1.24, 1.40, m	25.5	25.2
5	1.24,1.40, m	1.24, 1.40, m	31.9	31.7
6	3.24, m	3.24, m	73.1	72.6
7	3.35, m	3.35, m	72.7	72.4
8	2.18, m; 2.31, m	2.20, 2.33, m	37.0	36.7
9	6.59, dt, $J_{9,10}$ 15.89, $J_{9,8}$ 7.35	6.59, dt, $J_{9,10}$ 15.9, $J_{9,8}$ 7.5	152.3	151.7
10	5.75, d, $J_{10,9}$ 15.89	5.77, d, $J_{10,9}$ 15.9	108.5	108.0
11			77.0	76.5
12			60.3	59.7
13			72.3	71.8
14			66.0	65.4
15			68.3	67.7
16	3.93, s	3.99, s	74.5	74.0

*600 MHz for ^{1}H NMR
†^{13}C chemical shifts were assigned based on HSQC and HMBC

Supplementary Table 3. ^{1}H and ^{13}C NMR of massilin A 3 in C_2D_6OS

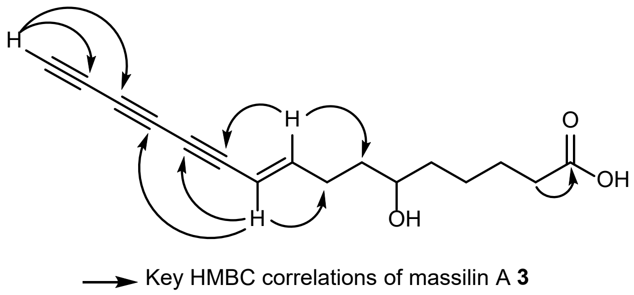

No.	δ_H (J in Hz)	δ_C
1	11.92, br s, COOH	174.4, COOH
2	2.18, t, $J_{2,3}$ 7.31	33.6
3	1.46, m	24.4
4	1.25, m; 1.35, m	24.6
5	1.28, m; 1.31, m	36.6
6	3.34, m	68.8
6-OH	4.43, d, $J_{OH,6}$ 5.50	
7	1.35, m; 1.42, m	35.6
8	2.18, m; 2.26, m	29.3
9	6.60, dt, $J_{9,10}$ 15.86, $J_{9,8}$ 7.13	153.8
10	5.75, d, $J_{10,9}$ 15.86	106.7
11		76.4
12		59.6
13		71.7
14		65.5
15		67.7
16	3.99, s	74.0

*600 MHz for ^{1}H NMR
†^{13}C chemical shifts were assigned based on HSQC and HMBC

Supplementary Table 4. ^{1}H and ^{13}C NMR of massilin B 4 in C_2D_6OS

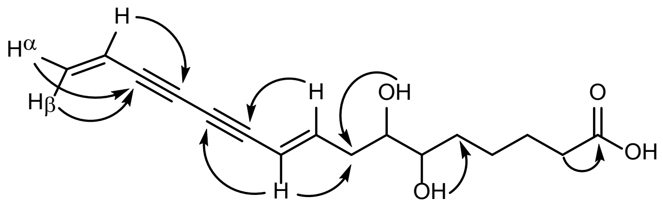

Key HMBC correlations of massilin B **4**

No.	δ_H (*J* in Hz)	δ_C
1	11.94, br s, COOH	174.4, COOH
2	2.19, t, $J_{2,3}$ 7.26	33.5
3	1.48, m	24.2
4	1.26,1.40, m	25.0
5	1.26,1.40, m	31.4
6	3.24, m	72.5
6-OH	4.37, d, $J_{OH,6}$ 5.92	
7	3.35, m	72.3
7-OH	4.53, d, $J_{OH,7}$ 6.0	
8	2.18, m; 2.31, m	36.2
9	6.43, dt, $J_{9,10}$ 15.8, $J_{9,8}$ 7.4	148.5
10	5.76, d, $J_{10,9}$ 15.8	108.8
11		81.5
12		71.8
13		74.3
14		79.8
15	6.04, dd, $J_{15,H\alpha}$ 11.38, $J_{15, H\beta}$17.55	115.8
16	H_α, 5.76, dd, $J_{H\alpha,15}$ 11.38, $J_{H\alpha, H\beta}$ 1.96<br>H_β, 5.85, dd, $J_{H\beta,15}$ 17.55, $J_{H\alpha, H\beta}$ 1.96	131.5

*600 MHz for ^{1}H NMR
†^{13}C chemical shifts were assigned based on HSQC and HMBC

Supplementary Table 5. ^{1}H and ^{13}C NMR of massilin C 5 in C_2D_6OS

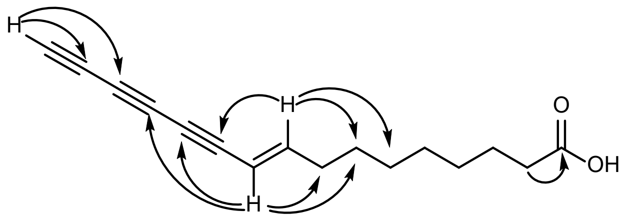

No.	δ_H (J in Hz)	δ_C
1		174.8, COOH
2	2.18, t, $J_{2,3}$ 7.36	33.7
3	1.48, m	24.8
4	1.24, m	28.7
5	1.24, m	28.7
6	1.24, m	28.7
7	1.34, m	27.7
8	2.15, m; 2.31, m	60.1
9	6.57, dt, $J_{9,10}$ 15.99, $J_{9,8}$ 7.10	153.9
10	5.75, d, $J_{10,9}$ 15.99	107.4
11		76.7
12		71.9
13		74.3
14		65.8
15		68.3
16	3.98, s	74.6

*600 MHz for ^{1}H NMR
†^{13}C chemical shifts were assigned based on HSQC and HMBC

Supplementary Table 6. Characterization of *mas* BGC and core genes in *ccn* BGC

Gene Name	Length (bp / aa)	Proposed Function	Similar Proteins	Sequence Identity (%aa)
masA	1080 / 359	Fatty acid desaturase	Fatty acid desaturase [*C. fungivorans* Ter331]<br>Fatty acid desaturase [*B. ambifaria* BCC0191], *ccnF*	70%<br>73%
masB	1005 / 334	Aromatic ring-hydroxylating dioxygenase subunit alpha	Rieske (2Fe-2S) domain protein [*C. fungivorans* Ter331]<br>Aromatic ring-hydroxylating dioxygenase subunit alpha [*B. ambifaria* BCC0191], *ccnG*	86%<br>83%
masC	822 / 273	Hypothetical protein	Hypothetical protein [*B. ambifaria* BCC0191], *ccnH*	72%
masD	1854 / 617	Fatty acyl-AMP ligase	Long chain fatty acid CoA ligase [*C. fungivorans* Ter331], *colA*<br>Fatty acyl-AMP ligase [*B. ambifaria* BCC0191], *ccnJ*	77%<br>52%
masE	903 / 300	Acyl-CoA desaturase	Delta-9 fatty acid desaturase [*C. fungivorans* Ter331], *colB*<br>Acyl-CoA desaturase [*B. ambifaria* BCC0191], *ccnK*	88%<br>58%
masF	981 / 326	Acyl-CoA desaturase	Delta-9 fatty acid desaturase [*C. fungivorans* Ter331], *colC*<br>Acyl-CoA desaturase [*B. ambifaria* BCC0191], *ccnL*	89%<br>75%
masG	330 / 109	Acyl carrier protein	Phosphopantetheine-binding protein [*C. fungivorans* Ter331], *colD*<br>Acyl carrier protein [*B. ambifaria* BCC0191], *ccnM*	94%<br>66%
masH	1101 / 365	Fatty acid desaturase	Fatty acid desaturase [*C. fungivorans* Ter331], *colE*<br>Fatty acid desaturase [*B. ambifaria* BCC0191], *ccnN*	88%<br>68%
masI	945 / 314	Alpha/beta hydrolase	Delta 12 desaturase [*C. fungivorans* Ter331], *colF*<br>Alpha/beta fold hydrolase [*B. ambifaria* BCC0191], *ccnO*	82%<br>52%
masJ	186 / 60	Rubredoxin	Rubredoxin-type Fe(Cys)4 protein [*C. fungivorans* Ter331]<br>Rubredoxin [*B. ambifaria* BCC0191], *ccnP*	86%<br>68%
masK	918 / 305	Alpha/beta hydrolase	None	-
masL	1182 / 393	Acetyl-CoA acetyltransferase	Acetyl-CoA acetyltransferase [*B. ambifaria* BCC0191]	59%
ccnD	1293 / 430	Beta-ketoacyl synthase	3-Oxoacyl-(acyl-carrier-protein) synthase [*C. fungivorans* Ter331]	75%
ccnE	1386 / 461	Flavin-dependent monooxygenase	Monooxygenase, FAD-binding protein [*C. fungivorans* Ter331]	88%
ccnI	1419 / 472	MFS_transporter	Drug transporter [*C. fungivorans* Ter331]	84%

Supplementary Table 7. The cell viabilities of *C. albicans* OE *masL* and OE *ERG10* under various polyynes.

	Mock	Collimonin C	Collimonin D	Massilin A	Massilin C
WT	100.1952	7.221861	6.050748	15.41965	6.942857
	100.7157	7.156799	7.156799	16.78595	6.342857
	99.21926	8.132726	6.636304	15.81002	6.685714
P_{tet}-*masL*$_{opt}$-His	99.40375	101.8952	99.72317	106.8143	5.916126
	98.76491	106.1116	99.14821	110.2002	5.691463
	97.23169	105.4089	97.87053	110.9668	6.215676
P_{tet}-*ERG10*	99.35856	66.70943	94.35536	53.55997	5.660377
	99.67928	67.41501	94.4195	58.62733	5.505299
	98.78127	75.75369	95.50994	61.19307	6.66839

Supplementary Table 8. List of primer sets used in this study

Primer name	Sequence (5'->3')
**Vector construction**	
masD-fF	AATGCCGACCCGGAGTTTCC
masD-fR	TCACACGGTGCCGCAGCTGATG
masE-fF	AAAGGTACCCCTGGCCTACATCACGGTGC
masE-fR	AAACTGCAGTCAGTGGTGGTTGTTGTGCCAGC
masF-fF	TGGGATTCGTGATCGCGGTG
masF-fR	CTAGTTGGCGCTGTTATCGTCCG
masH-fF	GCAAAAAGCTACCCATCATCGC
masH-fR	TCAGCCGAAGTAGACGATCGAGG
masI-fF	AAAGGTACCCCGGCATCGGCGCAT
masI-fR	AAACTGCAGTCACAGCAGGCGGTCGTAGAT
masJ-fF	AAAGGTACCGGTCAAGGAATTGATCACCCG
masJ-fR	TTCCACCATCGTGAAATCCTCTT
masJ-fF_point	CTGGCATAAACTGAAACCG
masJ-fR_point	CCGGTCAACCGTCCTTCTCG
masK-fF	TCTGGCGTTACTGCGGCTGG
masK-fR	ATGAGCTCATCTAGTCGAACGGCACGCGTTCG
masL-fF	AAAGGTACCACATTGAAGGACACTCCGGC
masL-fR	AAACTGCAGTCACGAGCTGACGATCATGGCGG
ERG10-F	GGAGCGGTCGACATCATTATGGTCCCACCTGTTTA
ERG10-R	GGAGCGAGATCTTTTGTCGTCCCCCTTACAATTTA
*ERG10*His-R	GAGCGAGATCTTAATGATGATGATGATGATGCAATTTAAAGTCACTATCGACTTTTT CAA
**Constructed strain examination**	
masD-F	GTCGTCGACCTGACCTATCGTC
masD-R	GACCTCCGGCTTGTTCAGGTAG
masE-F	CCCTCTTGCCACAGGAGTAA
masE-R	GTTTGCGTAGTCATGGCGTTT
masF-F	TCAAGCAGGTCACGGCGCTG
masF-R	TCGCCGAACGTCAGGATGGC
masH-F	CTCAACGCAACCGCCTAAAC
masH-R	TCAGGTATTCGGCACGGAGG
masI-F	TCTACGATCTCGATTCGGGC
masI-R	CTTCCGGTGAACCGTCCTTC
masJ-F	GTGCCGCTGAGCGAGG

Supplementary Table 8 (continued). List of primer sets used in this study

Primer name	Sequence (5'->3')
masJ-R	GCAGTAACGCCAGAATCAGGTTG
masK-F	TGTGCGGCTGGATCTATTCC
masK-R	CCCCATCACCACTTCGTCAA
masL-F	TGATCCCGCGCATCCTTAAC
masL-R	CAGCTTGATGGCCGGTACG
C_term-F	TTACCAATGCTTAATCAGTGAG
N_term-R	CGAACGACATGGAGCGGCAC
Location-check-F1	CCGAATTATTCCGGAAGCTGGTAGC
Location-check-R1	AAAGGGCAAAAGTGAGTATGGTGCC
Location-check-F2	GCCCATCAGAAACGACAAACATGGA
Location-check-R2	ACAATCAATGCCAGAGATCAAACCA
**Recombinant protein production**	
MasL-F	GGAATTCCATATGAAAGATGAAATCGTCATCAGTTC
MasL-R	CCCAAGCTTCACGCGTTCCAGCGCCAGCGCGATG
**Heterologous co-expression in *E. coli***	
*masB_Bam*HI_F	CGGGATCCGTACATCCAAGACGCATGGTAC
*masB_Nd*eI_F	GGAATTCCATATGTACATCCAAGACGCATGGTACGCAG
*masB_Sp*eI_*Hin*dIII_R	CCCAAGCTTACTAGTCAGACCGTCGCGCGGATGG
*masD_Eco*RI_F	GGAATTCATGACTTCCAGCTCGAGCATC
*masD_Sp*eI_*Hin*dIII_R	CCCAAGCTTACTAGTTACTCCTGTGGCAAGAGGGTG
*masE_Nd*eI_F	GGAATTCCATATGCAGGCGCAGAAGGATGGCGACAG
*masE_Sp*eI_*Hin*dIII_R	CCCAAGCTTACTAGTTATTTATTGATGGTTTCCGGTC
*masF_Nc*oI_F	CATGCCATGGATACTACGCAAACCAAGACCTTACCGCCCAAG
*masF_Sp*eI_*Hin*dIII_R	CCCAAGCTTACTAGTTATTGGGTCAGCGCGCGCTTGC
*masG_Nd*eI_F	GGAATTCCATATGTCTCAAGAAAACAAGGGAG
*masG_Sp*eI_*Hin*dIII_R	CCCAAGCTTGGCGGTTGCGTTGAGTCCGAG
*Bv4687_Nd*eI_F	GGGAATTCCATATGGCGAAAACCTACACGGTCCCCAG
*Bv4687*_R_*Sp*eI_*Hin*dIII	CCCAAGCTTACTAGTTCACCGGCCCTCCGCCGCCGCAC
*Bv4687_Bam*HI_F	CGGGATCCGGCGAAAACCTACACGGTCCCCAGCG
*masI*_F_*Nd*eI	GGAATTCCATATGACTGACATCACATCACCCTCG
*masI*_R_*Sp*eI_*Hin*dIII	CCCAAGCTTACTAGTCAGTCATTGCCAGATTCCAGTTC
*masJ*_F_*Nd*eI	GGAATTCCATATGACTGAAACCGTAGCTGAATACAAAAC
*masJ*_R_*Sp*eI_*Hin*dIII	CCCAAGCTTACTAGTCAGAATTCCACCATCGTGAAATCCTC

Supplementary Table 9. List of plasmids used in this study.

Plasmid	Description	Source
pCM184	Broad-host-range allelic exchange vector; Km^r, Tc^r, Ap^r	Mary Lidstrom Lab, addgene[9]
pCM184-Δ*masH*	pCM184-Δ*masH* for insertion on *masH* gene; Tc^r, Ap^r	This study
pNIM1	Plasmid for tetracycline-regulated gene expression system with GFP expression; Nc^r, Ap^r	Park and Morschhäuser[10]
pET-22b	Vector for construction C-terminal His-tagged recombinant protein; Ap^r	Novagen
pET-28a	Vector for construction N and C-terminal His-tagged recombinant protein; Km^r	Novagen
pNIM-*ERG10*	Tetracycline-inducible *CaERG10* overexpression plasmid derived from pNIM1 by replacing GFP gene; Nc^r, Ap^r	This study
pNIM-$ERG10_{\mathrm{L127S}}$	Tetracycline-inducible $CaERG10_{L127S}$ overexpression plasmid derived from pNIM1 by replacing GFP gene; Nc^r, Ap^r	This study
pNIM-$MasL_{\mathrm{opt}}$	Tetracycline-inducible $masL_{\mathrm{opt}}$ overexpression plasmid derived from pNIM1 by replacing GFP gene; Nc^r, Ap^r	This study
pET-22b-MasL	Recombinant protein production of *Massilia* sp. YMA4 MasL; Ap^r	This study
pET-22a(+)-*masD-masE-masF-masG*	IPTG inducible co-expression plasmid harboring *masD, masE*, *masF,* and *masG*. It was derived from pET-22a (+) vector. Ap^r	This study
pACYCduet-*Bv4687-masI-masJ*	IPTG inducible co-expression plasmid harboring *masH* homolog, *masI,* and *masJ.* It was derived from pACYCduet vector. Ch^r	This study
pACYCduet-*Bv4687-masI*	IPTG inducible co-expression plasmid, harboring *masH* homolog and *masI.* It was derived from pACYCduet. Ch^r	This study
pRSFduet-*masB.*	IPTG inducible co-expression plasmid of *masB.* It was derived from pRSFduet. Km^r	This study

*Km^r, Tc^r, Ap^r, Nc^r, and Ch^r indicate resistance to kanamycin, tetracycline, ampicillin, nourseothricin, and chloramphenicol.

Supplementary Table 10. List of strains used in this study

Strain	Description	Source
Massilia sp. YMA4	Wild-type strain; Km^r	This study
YMA4::*masD*	Biosynthesis mutant strain derived from *Massilia* sp. YMA4 by plasmid insertion at *masD* locus; Km^r, Tc^r	This study
YMA4::*masE*	Biosynthesis mutant strain derived from *Massilia* sp. YMA4 by plasmid insertion at *masE* locus; Km^r, Tc^r	This study
YMA4::*masF*	Biosynthesis mutant strain derived from *Massilia* sp. YMA4 by plasmid insertion at *masF* locus; Km^r, Tc^r	This study
YMA4::*masH*	Biosynthesis mutant strain derived from *Massilia* sp. YMA4 by plasmid insertion at *masH* locus; Km^r, Tc^r	This study
YMA4::*masI*	Biosynthesis mutant strain derived from *Massilia* sp. YMA4 by plasmid insertion at *masI* locus; Km^r, Tc^r	This study
YMA4::*masJ*	Biosynthesis mutant strain derived from *Massilia* sp. YMA4 by plasmid insertion at *masJ* locus; Km^r, Tc^r	This study
YMA4::*masK*	Biosynthesis mutant strain derived from *Massilia* sp. YMA4 by plasmid insertion at *masK* locus; Km^r, Tc^r	This study
YMA4::*masL*	Biosynthesis mutant strain derived from *Massilia* sp. YMA4 by plasmid insertion at *masL* locus; Km^r, Tc^r	This study
C. albicans ATCC18804	Wild-type strain	BCRC
P_{tet}-*ERG10*	*ERG10* overexpression strain derived from *C. albicans* ATCC18804; Nc^r	This study
P_{tet}-$ERG10_{L127S}$-His	*ERG10* overexpression strain derived from C. *albicans* ATCC18804 with L127S mutation and C-terminal 6xHis tag; Nc^r	This study
P_{tet}-$masL_{opt}$-His	$masL_{opt}$ with 6xHis tag heterologous expression strain derived from *C. albicans* ATCC18804; Nc^r	This study
E. coli S17-1 λ *pir*	*E. coli* donor strain for biparental conjugation	Prof. N.-C. Lin
E. coli C41(DE3)	*E. coli* used for recombinant protein production of *Massilia* sp. YMA4 MasL	Yeastern Biotech
E. coli DH5α	*E. coli* used for plasmid construction	Yeastern Biotech
mas-	*E. coli* C41 strain harboring *masD-masG*, *Bv4687*, *masI,* and *masJ*. Km^r, Ap^r	This study
mas-'	*E. coli* C41 strain harboring *masD-masG*, *Bv4687*, and *masI*. Km^r, Ap^r	This study
mas+	*E. coil* C41 strain harboring *masB, masD-masG, Bv4687*, *masI*, and *masJ*. Km^r, Ap^r, Ch^r	This study
mas+'	*E. coil* C41 strain harboring *masB, masD-masG, Bv4687,* and *masI*. Km^r, Ap^r, Ch^r	This study

*Km^r, Tc^r, Ap^r, Nc^r, and Ch^r indicate resistance to kanamycin, tetracycline, ampicillin, nourseothricin, and chloramphenicol.

Supplementary Methods

Chemicals, strains, plasmids, and culture conditions

ACS grade hexane (Hex), ethyl acetate (EA), dimethyl sulfoxide (DMSO); HPLC grade methanol (MeOH), isopropanol (IPA), and acetonitrile (ACN); and LCMS grade ACN were purchased from J. T. Baker (USA). Trifluoroacetic acid (TFA) and LCMS grade formic acid (FA) were purchased from Sigma-Aldrich (USA). Doxycycline was purchased from Cyrusbioscience, Inc. (Taiwan), and other antibiotics used in this study were purchased from Sigma (Sigma-Aldrich, USA). Culture media were purchased from BD Difco™ (USA). Coenzyme A was purchased from TRC (Toronto Research Chemicals, Canada). Acetyl-CoA and 7-diethylamino-3-(4-maleimidophenyl)-4-methylcoumarin (CPM) were purchased from Sigma-Aldrich (USA). Amphotericin B was purchased from Sigma-Aldrich (USA). Sequencing Grade Modified Trypsin was purchased from Promega (USA). All reagents used in crystallization were purchased from Hampton Research (USA).

The strains, plasmids, and primers used in this study are listed in **Supplementary Tables 8-10**. *Massilia* sp. YMA4 was isolated from a marine sediment core collected by research vessel Ocean Researcher No.3 at Lamay island offshore, Pingtung County, Taiwan, on November 9, 2013 (OR3-1727). The voucher specimen (BCRC 81003) was deposited in the Bioresource Collection and Research Center (BCRC), Food Industry Research and Development Institute, Taiwan. *Candida albicans* ATCC 18804 type strain (BCRC 20512) was purchased from BCRC. Plasmid pNIM1 and related materials were provided by Prof. Ching-Hsuan Lin, National Taiwan University[10]. *E. coli* S17-1 λ *pir*. is a generous gift from Prof. Nai-Chun Lin, National Taiwan University. All restriction enzymes and T4 DNA ligase were purchased from New England Biolabs (NEB, USA).

For strain maintenance, all *E. coli* strains were cultured in Luria-Bertani (LB) broth at 37 °C with the corresponding antibiotic supplement, *Massilia* sp. YMA4 was cultivated in yeast and malt extract broth (YMB) consisting of 3 g/L yeast extract; 3 g/L malt extract; 10 g/L Dextrose; 5 g/L Peptone, and 20 g/L Bacto agar (for solid medium) at 30 °C. *Candida* strains were cultured in yeast extract-peptone-dextrose medium (YPD) consisting of 10 g/L yeast extract, 20 g/L Peptone, 20 g/L Dextrose, and 20 g/L Bacto agar (for solid medium) at 30 °C.

RNA sequencing and transcriptomic analysis

Massilia sp. YMA4 was activated in 4 mL YMB for 24 hours, and 500 µL of activated *Massilia* sp. YMA4 broth was then transferred into 50 mL YMB in a 250 mL flask and cultured 24 hours. All the inoculated broth was cultured under 30 °C with 150 r.p.m. *Massilia* sp. YMA4 broth was then transplanted to YMA or PDA and cultured under 30 °C for 48 hours. *Massilia* sp. YMA4 cells were collected in TRIzol® reagent (Thermo Fisher Scientific, USA). The method of RNA extraction was followed the TRIzol® reagent protocol. First, the RNA extracts were treated with DNAse I for 15 minutes in ambient to clean up the genomic DNA, and then the purified total RNA was acquired using a MinElute PCR purification kit (Qiagen, Germany). Next, TruSeq Stranded Total RNA with Ribo-Zero kits (Illumina, USA) was used for mRNA-seq library preparation, then sequenced with paired-end reads (2 × 250 bp) using the Illumina MiSeq system. The raw-reads of RNA sequencing were deposited at the Sequencing Read Archive in the NCBI (National Center for Biotechnology Information) database under accession number PRJNA706894.

Construction of polyyne biosynthesis gene-null mutant strains

The DNA fragment containing a partial sequence of *masD*, *masE*, *masF*, *masH*, *masI, masJ, masK,* and *masL* were amplified with corresponding primers listed in **Supplementary Table 8**. The resulted PCR product was first cloned into yT&A vector (Yeastern Biotech, Taiwan) and subcloned into pCM184 by restriction-ligation at *Kpn*I/*Pst*I or *Eco*RI/ *Sac*I (only *masK*) site (New England Biolabs, USA) to generate pCM184-Δ*gene* plasmid (**Supplementary Figure 2** and **Supplementary Table 9**). For *masJ* mutant, after the homologous region constructing to pCM184, *masJ*-fF_point and *masJ*-fR_point primers were used to create point mutation on pCM184-Δ*masJ,* and the following steps were described above. The pCM184-Δ*gene* plasmid was further transformed into *E. coli* S17-1 for conjugation. The overnight culture of *Massilia* sp. YMA4 and *E. coli* S17-1 with pCM184-Δ*gene* were mixed with a 1:1 (v:v) ratio of optical density and further

cultured on YMA plates. The conjugants colonies were selected using oxytetracycline and kanamycin and checked by PCR with mutant examination primer sets (**Supplementary Figure 2** and **Supplementary Table 8**). The completed strains are listed in **Supplementary Table 10**.

UPLC-DAD-MS/MS methods

Approximately 5000 PDA agar plates were cultured with *Massilia* sp. YMA4 and extracted with EA 2-3 times. The extracts were concentrated and replaced with DMSO, preventing polyyne degradation while drying or resuspending for LC-MS/MS analysis.

All samples were adjusted to 10 mg/mL and analyzed by using an Agilent 1290 Infinity II ultra-performance liquid chromatography (UPLC) system coupled to an Agilent 1260 Infinity II DAD HS system and to a Dual AJS electrospray ionization (ESI) source of 6545XT AdvanceBio LC/Q-TOF (quadrupole time-of-flight) mass spectrometer (Agilent Technologies, USA). The chromatographic separation was performed on an ACQUITY BEH C18 UPLC column (2.1 × 100 mm, 1.7 μm; Waters, USA) with a 0.4 mL/min flow rate and 40 °C. Mobile phase A was 1‰ FA in water, and mobile phase B was 1‰ FA in ACN (LCMS grade). The gradient elution condition for polyyne profiling analysis was as follows: Initially, mobile phase B was held at 5% for 1 min, then changed from 5 to 100% linearly in 10 min. The mobile phase B was then held at 95% for 2 min. Finally, it decreased to 5% in 0.2 min and held on for 2.8 min. The gradient elution condition for collecting tandem mass spectra of polyynes was as follows: Initially, mobile phase B was held at 5% for 1min, followed by 5 to 30% linearly in 3 min, subsequent increased to 35% gradually from 4 to 8 min and then increased to 100% from 8 to 15 min. The mobile phase B was then held at 95% for 2 min. Finally, it decreased to 5% in 0.2 min and held on for 2.8 min. All LC-MS data used in this paper are publicly available at the GNPS-MassIVE repository under the accession number **MSV000087007**.

The diode array detector (DAD) was set as full-spectrum scanning with the UV range from 220 to 500 nm and scan rate 0.2 sec/spectrum. The electrospray ionization (ESI) source was used with negative ion mode. Data were collected in centroid mode with *m/z* 100–700 and scan rate 330 ms/spectrum for HRMS acquisition. For HR-MS/MS acquisition, automated data-dependent acquisition (DDA) mode was performed with *m/z* 100–700 and scan rate 330 ms/spectrum for precursor ion scanning. The top three selected precursor ions were fragmented with collision energy set at 10 eV and an isolation window of 1.3 Da. Data processing and peak identification were performed using Agilent MassHunter software (version B.08.00, Agilent Technologies, USA).

Construction of *mas* co-expression platform in *E. coli*

Individual *mas* genes were firstly cloned into the pET-28b(+) vector (Merck, Germany) using restriction enzymes *Nd*eI and *Hin*dIII, and additional *Sp*eI recognition sites were added to 3' terminal ends of genes. Next, the first gene was linked with the second gene by using complementary recognition sequences of *Sp*eI and *Xb*aI. To obtain the functional *masH* in the heterologous *E. coli* system, we have recruited native and codon-optimized *masH* and the homolog (*Bv4687*) from a palmitate-derived (C16) species *B. vietnamiensis* LMG 10929 (**Supplementary Figures 23, 29** and **40**) into modular co-expression. The backbone of constructed modules included pET-22a(+), pACYCduet, and pRSFduet were purchased from Merck (Germany). However, the polyyne accumulations were observed only in combination with *Bv4687*. Thus, *masH* was replaced by *Bv4687* in further co-expression assay. The pACYCduet-*Bv*4687-*masI* was built to investigate the possible role of *masJ* in polyyne biosynthesis.

Isolation, structure elucidation, and quantification of polyynes in *Massilia* sp. YMA4

In general, the EA extract of *Massilia* sp. YMA4 cultured on PDA plates was concentrated (not completely dried) and fractionated by Isolera One flash purification system (Biotage, Sweden). An HP silica column (20 μm, 12g, GRACE Inc., USA) was used with 12 mL/min flow rate. The solvent system consisted of A (Hex), B (EA) and C (MeOH) with gradient solvent elution programmed as fellows: 0-4 min, 100% A; 4- 20 min, 0-60% B; 20-23 min, 60-100% B; 23-30 min, 100% B; 30-35, 0-100% C; 35-42 min, 100% C. The polyyne fractions were monitored by UPLC-DAD-MS/MS and

combined as a polyyne-enriched fraction. The enriched fraction was further purified by reversed-phase-high performance liquid chromatography (RP-HPLC) on the Hitachi LaChrom Elite HPLC system with a Hitachi L-2130 pump and L-2455 Diode-array Detector (Hitachi, Japan). The RP-HPLC separation was performed using the Discovery HS C18 HPLC column (25 cm × 10 mm, 5 µm; SUPELCO Inc., USA) with a 4.25 mL/min flow rate at RT. The mobile phase A was 1‰ trifluoroacetic acid in water. The mobile B was 1‰ trifluoroacetic acid in acetonitrile (HPLC grade). The gradient elution condition for enriched fraction isolation was as follows: Initially, the concentration of B was changed from 20 to 44% linearly in 27 min, next increased to 100% immediately from 27 to 28 min. The ratio of mobile phase B was then held at 100% for 7 min. Finally, it decreased to 20% in 1 min and held on for 9 min to make a balance.

Collimonin C **1**, collimonin D **2,** and massilin B **4** were purified from polyyne enriched fractions by RP-HPLC. The RP-HPLC separation was performed on the Discovery HS C18 HPLC column (25 cm × 10 mm, 5 µm; SUPELCO Inc., Missouri, US) with a flow rate of 3.25 mL/min at RT. The mobile phase A was 1‰ trifluoroacetic acid in water. The mobile B was 1‰ trifluoroacetic acid in acetonitrile/isopropanol (3:7, HPLC grade). The gradient elution condition for enriched fraction isolation was as follows: Initially, the concentration of B was changed from 30 to 42.5% linearly in 25 min, following an increase to 100% from 25 to 30 min. The ratio of mobile phase B was then held at 100% for 5 min.

For further purification of massilin A **3**, the RP-HPLC separation was performed on the Discovery HS C18 HPLC column (25 cm × 10 mm, 5 µm; SUPELCO Inc., US) with a flow rate of 3.25 mL/min at RT. The mobile phase A was 1‰ trifluoroacetic acid in water. The mobile B was 1‰ trifluoroacetic acid in acetonitrile/isopropanol (3:7, HPLC grade). The gradient elution condition for enriched fraction isolation was as follows: Initially, the concentration of B was changed from 38.5 to 51% linearly in 25 min, subsequently increased to 100% from 25 to 30 min. The ratio of mobile phase B was then held at 100% for 5 min.

Massilin C **5** was purified from heterologous co-expression of *mas* genes (*masD- masJ*) in the *E. coli* strain (*mas*-). The EA extract was concentrated into DMSO and purified by RP-HPLC (Experimental procedure 5). The RP-HPLC separation was performed on the Discovery HS C18 HPLC column (25 cm × 10 mm, 5 µm; SUPELCO Inc., Missouri, US) with a flow rate of 3.25 mL/min at RT. The mobile phase A was 1‰ trifluoroacetic acid in water. The mobile B was 1‰ trifluoroacetic acid in acetonitrile (HPLC grade). The gradient elution condition for enriched fraction isolation was as follows: Initially, the concentration of B was isocratic at 65% in 14 min, following an increase to 100% from 14 to 15 min. The ratio of mobile phase B was then held at 100% for 5 min.

All the purified polyynes were stored in methanol. Polyynes for all experiments were prepared freshly, and the solvent was replaced by dimethyl sulfoxide-d_6 (C_2D_6OS, 99.9% D; Cambridge Isotope Laboratories, Inc., USA) for the following experiments. The physicochemical properties of collimonin C **1** and collimonin D **2** were identical with the report from *Collimonas fungivorans* Ter331[2]. The UV absorption at 272, 288, 307, 329 nm suggests that collimonin C **1**, collimonin D **2**, massilin A **3**, and massilin C **5** were functionalized with enetriyne moiety. The UV absorption at 274, 290, 310 nm suggests massilin B **4** was functionalized with enediyne-ene moiety. The NMR data of collimonin C **1** and collimonin D **2** were identical to previous published data [2].

The molecular formula of Massilin A **3** was determined as $C_{16}H_{18}O_3$ by HRESIMS with *m/z* 257.1189 $[M-H]^-$ (calcd. for $C_{16}H_{17}O_3$, 257.1183). The ^1H NMR data accounted for 16 non-exchangeable protons, composed of one terminal alkyne proton, two (*E*)-olefinic protons (J = 15.86 Hz), one oxymethine, and six methylene groups (**Supplementary Table 3** and **Supplementary Figure 14**). Analysis of HSQC and HMBC spectra accounted for 16 carbon signals, and the presence of one carboxyl group, one (*E*)-olefin, one triyne, one oxymethine, and six methylene groups were identified (**Supplementary Figures 16-17**). Through the ^1H-^1H COSY and HMBC correlations, the structure of **3** was determined as a hexadecanoic acid derivative possessing one enetriyne moiety and one hydroxyl group at C-6 (**Supplementary Table 3**, **Supplementary Figures 15** and **17**).

The molecular formula of Massilin B **4** was determined as $C_{16}H_{20}O_4$ by HRESIMS with *m/z* 275.1298 $[M-H]^-$ (calcd. for $C_{16}H_{19}O_4$, 275.1289). The ^1H NMR data accounted for 17 non-exchangeable protons, composed of one terminal alkene group, two (*E*)-olefinic protons (J = 15.8

Hz), two oxymethines, and five methylene groups (**Supplementary Table 4** and **Supplementary Figure 18**). Analysis of HSQC and HMBC spectra accounted for 16 carbon signals, and the presence of one carboxyl group, one (*E*)-olefin, one terminal alkene, one diyne, two oxymethines, and five methylene groups were identified (**Supplementary Figures 20-21**). Through the ^{1}H-^{1}H COSY and HMBC correlations, the structure of **4** was determined as a hexadecanoic acid derivative possessing one enediyne-ene moiety and two hydroxyl groups at C-6 and C-7, respectively (**Supplementary Table 4** and **Supplementary Figures 19** and **21**).

The molecular formula of Massilin C **5** was determined as $C_{16}H_{18}O_2$ by HRESIMS with *m/z* 243.1236 [*M*-H]$^-$ (calcd. for $C_{16}H_{17}O_2$, 243.1234). The ^{1}H NMR data accounted for 17 non-exchangeable protons, composed of one terminal alkyne proton, two (*E*)-olefinic protons (J = 15.99 Hz), and seven methylene groups (**Supplementary Table 5** and **Supplementary Figure 25**). Analysis of HSQC and HMBC spectra accounted for 16 carbon signals, and the presence of one carboxyl group, one (*E*)-olefin, one triyne, and seven methylene groups were identified (**Supplementary Figures 27-28**). Through the ^{1}H-^{1}H COSY and HMBC correlations, the structure of **5** was determined as a hexadecanoic acid derivative possessing one enetriyne moiety (**Supplementary Table 5**, **Supplementary Figures 26** and **28**).

NMR spectra were recorded on a Bruker Ascend 600 NMR spectrometer with a Prodigy cryoprobe using C_2D_6OS. NMR spectra were processed using Bruker Topspin (version 3.6) and MestReNova (Mestrelab, version 14.0.0). Spectra were referenced to residual solvent signals with resonances at δ_H 2.50 for dimethyl sulfoxide. The ^{13}C chemical shifts of all polyynes were assigned based on HSQC and HMBC. The quantitation of polyynes was measured in ^{1}H NMR using the ^{13}C-coupling satellite peak of C_2D_6OS (~1.1% of ^{12}C; 2.39/2.61)[11] with an equation **(1)**:

$$\boldsymbol{C_{polyyne}} = \frac{\boldsymbol{I_{polyyne}}}{\boldsymbol{I_{[D_6]DMSO}}} \times \frac{\boldsymbol{N_{[D_6]DMSO}}}{\boldsymbol{N_{polyyne}}} \times \boldsymbol{C_{[D_6]DMSO}} \quad \textbf{(1)}$$

C is the concentration, *I* is integral of proton signal, and *N* is the number of nuclei giving rise to the signal. The purity was measured using HPLC-DAD with the same gradient condition for purification at 241 nm with over 95% purity (integrated area).

Collimonin C 1: light yellow dissolved in DMSO; ^{1}H and ^{13}C NMR (**Supplementary Table 1**, C_2D_6OS, 256 K); HRMS(ESI$^-$) *m/z* 273.1138 [*M*-H]$^-$ (calcd. for $C_{16}H_{17}O_4$, 273.1132).
Collimonin D 2: light yellow dissolved in DMSO; ^{1}H and ^{13}C NMR (**Supplementary Table 2**, C_2D_6OS, 256 K); HRMS(ESI$^-$) *m/z* 273.1139 [*M*-H]$^-$ (calcd. for $C_{16}H_{17}O_4$, 273.1132).
Massilin A 3: racemate, light yellow dissolved in DMSO; ^{1}H and ^{13}C NMR (**Supplementary Table 3**, C_2D_6OS, 256 K); HRMS(ESI$^-$) *m/z* 257.1189 [*M*-H]$^-$ (calcd. for $C_{16}H_{17}O_3$, 257.1183).
Massilin B 4: light yellow dissolved in DMSO; ^{1}H and ^{13}C NMR (**Supplementary Table 4**, C_2D_6OS, 256 K); HRMS(ESI$^-$) *m/z* 275.1298 [*M*-H]$^-$ (calcd. for $C_{16}H_{19}O_4$, 275.1289).
Massilin C 5: light yellow dissolved in DMSO; ^{1}H and ^{13}C NMR (**Supplementary Table 5**, C_2D_6OS, 256 K); HRMS(ESI$^-$) *m/z* 243.1236 [*M*-H]$^-$ (calcd. for $C_{16}H_{17}O_2$, 243.1234).
The absolute configuration of collimonin C **1** and D **2** was confirmed by Mosher ester analysis and compared to reported chemical shifts and coupling constants.

Construction of inducible *ERG10* overexpression strains and *masL*$_{opt}$ heterologous expression strain in *C. albicans*

ERG10 fragment was amplified with *ERG10*-F, *ERG10*-R, and *ERG10His*-R. The *masL*$_{opt}$ fragment was optimized and synthesized by BIOTOOLS (Taiwan). The fragments were respectively cloned into the pNIM1 vector for creating pNIM-*ERG10*, pNIM-*ERG10*$_{L127S,}$ and pNIM-*MasL*$_{opt}$ plasmids (**Supplementary Table 9**). The expression module was first linearized by *Apa*I/*Sac*II digestion and transformed into *C. albicans* ATCC18804 using lithium acetate. Then, the inducible *EGR10* overexpression strains and the *masL*$_{opt}$ heterologous expression strain were generated by replacing the single allele of *ADH1* with the linearized fragments (**Supplementary Figure 41** and **Supplementary Table 10**). Finally, the completed strains were selected on yeast extract-peptone-

dextrose (YPD) plates with nourseothricin (Werner Bioagents, Jena, Germany) according to the previous research[10].

Transmission Electron Microscope

C. albicans was cultured in YPD started with OD_{600} = 0.05 at 37 °C for 2 hours. The broth cultures were independently treated with 1% ethyl acetate (EA), 1 mg/mL Δ*masH* crude extract (in 1% EA), and 1 mg/mL *Massilia* sp. YMA4 crude extract (in 1% EA) and then cultured at 37 °C for 4 hours. All samples were obtained and fixed using 0.1 M phosphate buffer, followed by a similar fixation process in a previous study[12]. Finally, the samples were embedded in pure Spurr's resin (Sigma-Aldrich, USA), processed with ultrathin section, and observed under an FEI Tecnai™ G^2 F20 S-TWIN transmission electron microscope (FEI Company, USA).

Construction of inducible *masL* heterologous expression strain in *E. coli*

The gene *masL* from *Massilia* sp. YMA4 was cloned into the site between *Nde*I/*Xho*I of vector pET-22b(+) by In-Fusion cloning (Takara Bio, USA). The plasmid pET-22b-MasL was transformed into *E. coli* C41(DE3) (Yeastern Biotech, Taiwan) via heat shock and selected with ampicillin and kanamycin, respectively, for overexpressing recombinant hexahistidine-tagged proteins.

Expression and purification of MasL and ERG10

For the expression of MasL, an overnight culture of *E. coli* in LB, supplemented with appropriate antibiotics, was then used to inoculate 1 L 2x YT broth (16 g/L Tryptone; 10 g/L Yeast extract; 5 g/L NaCl) and incubated at 37 °C, 200 rpm to OD_{600} = 0.4 - 0.6. Protein expression was induced upon adding 0.5 mM IPTG for 16 h at 16 °C, 200 rpm. The cells were harvested via centrifugation (4000 × g, 4 °C, 15 min) and resuspended lysis buffer (50 mM Tris·HCl pH 8.5, 500 mM NaCl, 30 mM imidazole (Merck, Germany), and 300 µg/mL lysozyme (Sigma-Aldrich, USA) for cell lysing for 30 min at 37 °C. The lysed cells were further disturbed using Misonix Sonicator XL2020 (Misonix, USA) and centrifuged (10,000 × g, 4 °C, 30 min).

For the expression of $ERG10_{L127S}$, an overnight culture of P_{tet}-*ERG10*$_{L127S}$-*His* in YPD, supplemented with nourseothricin, was used to inoculate in 4 L YPD broth at 37 °C, 200 rpm to OD_{600} = 0.4 - 0.6. Protein expression was induced upon adding 40 mM doxycycline for 16 h at 30 °C, 200 rpm. The cells were harvested via centrifugation (4000 × g, 4 °C, 15 min) and resuspended in lysis buffer (50 mM Tris·HCl pH 8.5, 500 mM NaCl and 30 mM imidazole), followed by lysing using a French press operated at 4 °C (20 kpsi) (Constant System RCB411, Constant Systems, USA) and centrifuged (10,000 × g, 4 °C, 30 min).

The first affinity purification was conducted with a 5 mL HisTrap HP column (GE Healthcare Bio-Sciences, USA) pre-equilibrated with lysis buffer and washed with three column volumes (CV) of wash buffer (50 mM Tris·HCl pH 8.5, 500 mM NaCl, and 30 mM imidazole), and eluted with 20 CV elution buffer with a gradient concentration of imidazole (50 mM Tris·HCl pH 8.5, 500 mM NaCl, with imidazole from 50 to 500 mM). The eluents were ultra-filtrated and buffer-exchanged to gel-filtration buffer (20 mM Tris·HCl pH8.5, 100 mM NaCl) using Amicon Ultra-15 centrifugal filter units (Merck, Germany) with a 30 kDa cutoff (MWCO). The His-tag purified proteins were further loaded on Superdex 200 Increase 10/300 GL column (GE Healthcare Bio-Sciences, USA) for size-exclusion chromatography with gel-filtration buffer (20 mM Tris·HCl pH8.5, 100 mM NaCl) in the isocratic method. The eluents were subjected to SDS-PAGE analysis. Target protein-contained eluents were combined, ultra-filtrated, and then added glycerol to a final 10% for storage and further crystallization and enzyme assay. All chromatography was performed on ÄKTA pure 25 M1 purification system (GE Healthcare Bio-Sciences, USA). The concentration of purified protein was measured using absorbance at 280 nm by NanoDrop 1000 Spectrophotometer (Thermo Fisher Scientific, USA) with calculated Extinction coefficients (ε)[13]: MasL (20970 M^{-1} cm^{-1}) and $ERG10_{L127S}$ (15930 M^{-1} cm^{-1}).

Enzymatic inhibition assay and inhibition kinetics of polyynes

The enzymatic inhibition assay was initiated by adding 50 μM polyynes (collimonin C **1**, collimonin D **2,** and massilin A **3**) into 10 μM $ERG10_{L127S}$ at 25 °C for 1 h. The residue active enzyme reaction started by adding 10 mM acetyl-CoA for another 1 h at 25 °C in a total of 12 μL volume with the following concentrations: 8.33 μM enzymes, 41.65 μM polyynes, and 1.67 mM acetyl-CoA in reaction buffer (20 mM Tris·HCl pH8.5, 100 mM NaCl). The reaction was quenched by adding 1 μL of 1% FA (final ~pH 2.5). The monitor method of releasing CoA using a fluorescent probe (7-diethylamino-3-(4-maleimidophenyl)-4-methylcoumarin, CPM) was modified from previous research[14]. The released CoA was used to represent the residual activity or protein occupancy. After 10 min, the pH value was adjusted by using 2 μL 0.1 M Tris pH10 (final ~pH 7.5) and 100 μM CPM probe was added in a total volume of 105 μL for 30 min reaction at 30°C followed by detection of the fluorescent signal using a BioTek Synergy H1 microplate reader (excitation 355 nm; emission 460 nm) (BioTek, USA). Relative fluorescence intensity was obtained by subtracting the fluorescence intensity of the polyyne-free reaction system.

Protein occupancy and inhibition kinetic calculations were as the following equation:

$$\textbf{Total occupancy} = [\mathbf{E}-\mathbf{I}]/[\mathbf{E}]_{\mathbf{int}} = 1-([\mathbf{E}]_{\mathbf{free}}/[\mathbf{E}]_{\mathbf{int}}) = 1-e^{-(k_{obs}*t_I)} \quad \textbf{(2)}$$

$$k_{obs} = \frac{k_{inact}[\textbf{Inhibitor}]}{\mathbf{K_I} + [\textbf{Inhibitor}]} \quad \textbf{(3)}$$

The observed rate constant for inhibition (k_{obs}) is a pseudo-first-order rate constant obtained from the product formation by fitting the kinetic data to equation **(2)**. For irreversible covalent inhibitors, kinetic parameters k_{inact} (rate of inactivation, maximum k_{obs} at infinite inhibitor concentration) and K_I (concentration inhibitor that yields a half-maximum k_{obs}; ½ k_{inact}) can be obtained from plotting k_{obs} values as a function of [inhibitor], and fitting to equation **(3)**.

Protein crystallization, data collection, processing, and refinement

For MasL-collimonin C and MasL-collimonin D complex preparation, 20 μM MasL was incubated with 100 μM collimonin C **1** and collimonin D **2** in 20 mM Tris-HCl pH 8.5 and 100 mM NaCl, separately. The MasL-collimonin C and MasL-collimonin D complex was purified with a gel-filtration (Superdex 200 Increase 10/300 column).

A freshly thawed aliquot of MasL, MasL-collimonin C complex, and MasL-collimonin D complex was concentrated to 20 mg/mL for an initial crystallization screening of ca. 500 conditions (Academia Sinica Protein Clinic, Academia Sinica). The crystallization conditions were manually refined to the final conditions: for MasL, 2% (v/v) Tacsimate pH 7.0, 16% (w/v) polyethylene glycol 3,350, and 0.1 M HEPES, pH 7.5; for MasL-collimonin C and MasL-collimonin D complex, 20% (w/v) polyethylene glycol 3,350 and 0.2 M tri-lithium citrate, pH 8. The crystals were grown at 10 °C by mixing the protein aliquot with an equivalent volume of crystallization buffer via the hanging drop vapor diffusion method. The crystals were immediately flash-frozen in liquid nitrogen for X-ray data collection after dipping into cryoprotectant composed of crystallization solution supplemented with 10% (v/v) glycerol.

X-ray diffraction experiments were conducted at 100 K at the TLS beamline 15A or the TPS beamline 05A of the National Synchrotron Radiation Research Center (Hsinchu, Taiwan) with a wavelength of 1 Å. All diffraction data were processed and scaled with the HKL-2000 package[15]. The data collection statistics are listed in **Table 2**.

The MasL, MasL-collimonin C, and MasL-collimonin D complex structures were solved by the molecular replacement method with the program Molrep[16] using the structure of thiolase from *Clostridium acetobutylicum* (pdb ID **4N44**) as the search model. Computational model building was conducted with ARP/wARP or Buccaneer[17,18,] and the rest of the models were manually built with Coot[19]. Finally, the resulting models were subjected to computational refinement with Refmac5[20].

The collimonin C **1**, collimonin D **2,** and well-ordered water molecules were located with Coot. The stereochemical quality of the refined models was checked with MolProbity[21]. Finally, the MasL, MasL-collimonin C, and MasL-collimonin D complex's refinement converged at a final R factor/R_{free} of 0.128/0.180, 0.114/0.162, and 0.146/ 0.163, respectively. The final refinement statistics are listed in **Table 2**.

Sequence alignment and structure superimposition

The protein sequence alignment was performed in the CLC genomics workbench (version 11, CLC bio, Denmark) with gap open cost 10.0 and extension cost 1.0. The protein superimposition was processed and produced figures in Maestro (Schrödinger Release 2021-1: Maestro, Schrödinger, USA).

Bottom-up mass spectrometry analysis and peptide mapping of polyyne-labeled peptides

Incubation mixture (20 µL) containing 2 µM recombinant protein (MasL or $ERG10_{L127S}$ in 50 mM Tris pH 8.5, 100 mM NaCl) was incubated with 40 µM collimonin C **1**, collimonin D **2**, or massilin A **3** at 25 °C for 3 h. The reaction was quenched by adding 4x Laemmli sample buffer (Bio-Rad, USA) with 5 mM DL-dithiothreitol, and the protein was separated using SDS-PAGE. The in-gel trypsin digestion was performed with a substrate-to-enzyme ratio of 25:1 (w/w), and the mixture was incubated at 37 °C for 20 h[22]. The resultant peptide mixtures were dried and frozen at -20 °C until analysis using nanoUPLC-ESI-MS. The tryptic peptides were re-dissolved in 10 µL of 0.1% formic acid. An LC-nESI-Q Exactive mass spectrometer (Thermo Scientific, USA) coupled with an online nanoUPLC (Dionex UltiMate 3000 Binary RSLCnano, USA) was used for analysis. An Acclaim PepMap 100 C18 trap column (75 µm x 2.0 cm, 3 µm, 100 Å, Thermo Scientific, USA) and an Acclaim PepMap RSLC C18 nanoLC column (75 µm x 25 cm, 2 µm, 100 Å) were used with a linear gradient from 5% to 35% of acetonitrile in 0.1% (v/v) formic acid for 40 min at a flow rate of 300 nL/min. The MS data were collected in the data-dependent acquisition mode with a full MS scan followed by 10 MS/MS scans of the top 10 precursor ions from the full MS scan. The MS scan was performed with 70,000 resolution over *m/z* range 350 to 1600, and dynamic exclusion was enabled. The data-dependent MS/MS acquisition was performed with a two *m/z* isolation window, 27% normalized collision energy and 17,500 resolution.

The data were processed using Proteome Discoverer (version 2.4, Thermo Scientific, USA). The peptides were identified by searching the MS/MS spectra against the MasL and $ERG10_{L127S}$ using the Mascot search engine (version 2.3, Matrix Science, UK) and SEQUEST search engine[23]. Cysteine alkylation was used as a dynamic modification, and the modification *m/z* values were +274.121 (+$C_{16}H_{18}O_4$ for collimonin C/D **1, 2**) and +258.126 (+$C_{16}H_{18}O_3$ for massilin A **3**), respectively. For identification, the false discovery rate was set to 0.01 for peptides, proteins, and sites. The minimum peptide length allowed was four amino acids, precursor mass tolerance for 10 ppm, and fragment mass tolerance for 0.02 Da. The raw data from the bottom-up proteomics analysis are publicly available at the GNPS-MassIVE repository under the MassIVE accession number **MSV000087027**.

SI References

1. Kumar, S., Stecher, G., Li, M., Knyaz, C. & Tamura, K. MEGA X: Molecular evolutionary genetics analysis across computing platforms. *Mol. Biol. Evol.* **35**, 1547-1549 (2018).
2. Kai, K., Sogame, M., Sakurai, F., Nasu, N. & Fujita, M. Collimonins A–D, unstable polyynes with antifungal or pigmentation activities from the fungus-feeding bacterium *Collimonas fungivorans* Ter331. *Org. Lett.* **20**, 3536-3540 (2018).
3. Parker, W. L. *et al.* Cepacin A and cepacin B, two new antibiotics produced by *Pseudomonas cepacia*. *J. Antibiot. (Tokyo)* **37**, 431-440 (1984).
4. Mullins, A. J. *et al.* Genome mining identifies cepacin as a plant-protective metabolite of the biopesticidal bacterium *Burkholderia ambifaria*. *Nat. Microbiol.* **4**, 996-1005 (2019).
5. Ross, C., Scherlach, K., Kloss, F. & Hertweck, C. The molecular basis of conjugated polyyne biosynthesis in phytopathogenic bacteria. *Angew. Chem. Int. Ed. Engl.* **53**, 7794-7798 (2014).
6. Murata, K., Suenaga, M. & Kai K. Genome mining discovery of Protegenins A–D, bacterial polyynes involved in the antioomycete and biocontrol activities of *Pseudomonas protegens*. *ACS Chem. Biol*; 10.1021/acschembio.1c00276 (2021).
7. Mullins, A. J. *et al*. Discovery of the *Pseudomonas* polyyne Protegencin by a phylogeny-guided study of polyyne biosynthetic gene cluster diversity. *mBio* **12**, e0071521 (2021).
8. Zhang, Y. *et al. Aspergillus fumigatus* mitochondrial acetyl coenzyme A acetyltransferase as an antifungal target. *Appl. Environ. Microbiol.* **86**, e02986-19 (2020).
9. Marx, C. J.& Lidstrom M. E. Broad-host-range cre-lox system for antibiotic marker recycling in gram-negative bacteria. *Biotechniques* **33**, 1062-1067 (2002).
10. Park, Y. -N. & Morschhäuser, J. Tetracycline-inducible gene expression and gene deletion in *Candida albicans*. *Eukaryot. Cell* **4**, 1328-1342 (2005).
11. Moutzouri, P. *et al*. (13)C Satellite-Free (1)H NMR Spectra. *Anal. Chem.* **89**, 11898-11901 (2017).
12. Vazquez-Muñoz, Avalos-Borja, R. M. & Castro-Longoria, E. Ultrastructural analysis of *Candida albicans* when exposed to silver nanoparticles. *PLoS One* **9**, e108876 (2014).
13. Wilkins, M. R. *et al.* Protein identification and analysis tools in the ExPASy server in *Methods in Molecular Biology* (ed. Link, A. J.) 531-552 (Humana Press, 1999).
14. Long, T., Sun, Y., Hassan, A., Qi, X. & Li, X. Structure of nevanimibe-bound tetrameric human ACAT1. *Nature* **581**, 339-343 (2020).
15. Otwinowski, Z. & Minor, W. Processing of X-ray diffraction data collected in oscillation mode. *Methods Enzymol.* **276**, 307-326 (1997).
16. Vagin, A. & Teplyakov, A. Molecular replacement with MOLREP. *Acta Crystallogr. D Biol. Crystallogr.* **66**, 22-25 (2010).
17. Cowtan, K. The Buccaneer software for automated model building. 1. Tracing protein chains. *Acta Crystallogr. D Biol. Crystallogr.* **62**, 1002-1011 (2006).
18. Perrakis, A., Morris, R. & Lamzin, V. S. Automated protein model building combined with iterative structure refinement. *Nat. Struct. Biol.* **6**, 458-463 (1999).
19. Emsley, P. & Cowtan K. Coot: model-building tools for molecular graphics. *Acta Crystallogr. D Biol. Crystallogr.* **60**, 2126-2132 (2004).
20. Murshudov, G. N. *et al.* REFMAC5 for the refinement of macromolecular crystal structures. *Acta Crystallogr. D Biol. Crystallogr.* **67**, 355-367 (2011).
21. Williams, C. J. *et al*. MolProbity: More and better reference data for improved all-atom structure validation. *Protein Sci.* **27**, 293-315 (2018).
22. Shevchenko, A., Tomas, H., Havlis, J., Olsen, J. V. & Mann, M. In-gel digestion for mass spectrometric characterization of proteins and proteomes. *Nat Protoc.* **1**, 2856-2860 (2006).
23. Diament, B. J. & Noble, W. S. Faster SEQUEST searching for peptide identification from tandem mass spectra. *J. Proteome Res.* **10**, 3871-3879 (2011).
