# Supplementary material for: Integrated omics approach to unveil antifungal bacterial polyynes as acetyl-CoA acetyltransferase inhibitors
Source: Commun Biol. 2022 May 12;5:454. doi: 10.1038/s42003-022-03409-6 (PMC9098870; doi:10.1038/s42003-022-03409-6)
Supplement: Supplementary file 3 — Description of Additional Supplementary Files [file 42003_2022_3409_MOESM3_ESM.pdf]

Description of Additional Supplementary Files

File name: Supplementary Data 1

Description: Transcriptome analysis for different culture media

File name: Supplementary Data 2

Description: Multigeneblast result of massilin BGC query in the BCT database

File name: Supplementary Data 3

Description: Bottom up proteome result for polyyne-modification protein
