# Supplementary material for: Integrated omics approach to unveil antifungal bacterial polyynes as acetyl-CoA acetyltransferase inhibitors
Source: Commun Biol. 2022 May 12;5:454. doi: 10.1038/s42003-022-03409-6 (PMC9098870; doi:10.1038/s42003-022-03409-6)
Supplement: Supplementary file 7 — Reporting Summary [file 42003_2022_3409_MOESM7_ESM.pdf]

nature portfolio

Corresponding author(s): Yu-Liang Yang

Last updated by author(s): Mar 10, 2022

Reporting Summary

Nature Portfolio wishes to improve the reproducibility of the work that we publish. This form provides structure for consistency and transparency in reporting. For further information on Nature Portfolio policies, see our Editorial Policies and the Editorial Policy Checklist.

Statistics

For all statistical analyses, confirm that the following items are present in the figure legend, table legend, main text, or Methods section.

n/a	Confirmed	
☐	☒	The exact sample size (*n*) for each experimental group/condition, given as a discrete number and unit of measurement
☐	☒	A statement on whether measurements were taken from distinct samples or whether the same sample was measured repeatedly
☐	☒	The statistical test(s) used AND whether they are one- or two-sided<br>*Only common tests should be described solely by name; describe more complex techniques in the Methods section.*
☒	☐	A description of all covariates tested
☒	☐	A description of any assumptions or corrections, such as tests of normality and adjustment for multiple comparisons
☐	☒	A full description of the statistical parameters including central tendency (e.g. means) or other basic estimates (e.g. regression coefficient) AND variation (e.g. standard deviation) or associated estimates of uncertainty (e.g. confidence intervals)
☐	☒	For null hypothesis testing, the test statistic (e.g. *F*, *t*, *r*) with confidence intervals, effect sizes, degrees of freedom and *P* value noted<br>*Give P values as exact values whenever suitable.*
☒	☐	For Bayesian analysis, information on the choice of priors and Markov chain Monte Carlo settings
☒	☐	For hierarchical and complex designs, identification of the appropriate level for tests and full reporting of outcomes
☒	☐	Estimates of effect sizes (e.g. Cohen's *d*, Pearson's *r*), indicating how they were calculated

Our web collection on statistics for biologists contains articles on many of the points above.

Software and code

Policy information about availability of computer code

Data collection	MassHunter Acquisition software (version B.08.00, Agilent Technologies, USA) was used to collect mass spectrometry data.<br>Illumina MiSeq system (Illumina, USA) was used to collect RNA-seq data.<br>Epoch 2 Microplate (BioTek Instruments, USA) was used to collect optical density data.<br>MXCuBE (version 2.0) was used to collect X-ray diffraction raw data.<br>Bruker Topspin (version 3.6) was used to collect vuclear magnetic resonance spectroscopy data.<br>Tecnai User Interface (FEI Company, USA) was used to collect transmission electron microscope imaging data.<br>ND-1000 spectrophotometer software (version 3.8.1) was used to collect UV absorbance spectra data.<br>BioTek Gen5 (BioTek, USA) was used to collect fluorescence spectroscopy for data.<br>Xcalibur™ Software(version 4.3, Thermo Scientific, USA) was used to collect mass spectrometry data.
Data analysis	DeepBGC (version.23) for BGC prediction analysis; MultiGeneBlast for BGC detection/alignment analysis; MEGA (version 10) for phylogenetical analysis; iTOL (web) for phylogenetical result visualization; CLC genomics workbench (version 11, CLC bio, Denmark) for Miseq FastQ file analysis; GraphPad Prism (Version 8, GraphPad Software, USA) for statistics; Maestro (Schrödinger Release 2021-1: Maestro, Schrödinger, USA) for protein structure model visualization; HKL-2000 (HKL Research, Virginia) for X-ray diffraction data pre-processing; MassHunter Qualitative workflow software (version B.08.00, Agilent Technologies, USA) for small molecular mass spectrometry data processing; MestReNova (Mestrelab, version 14.0.0) for NMR spectra processing; Molrep (version 7.3), ARP/wARP (version 8.0), Buccaneer (version 1.1.9), Coot(version 6.1), Refmac (version 5.0.32), MolProbity in CCP4 (version 7.2) for crystal model building and refinement; Proteome Discoverer (version 2.4, Thermo Scientific, USA) for proteomic analysis; Mascot search engine (version 2.3, Matrix Science, UK) for peptide in-silico library searching.

For manuscripts utilizing custom algorithms or software that are central to the research but not yet described in published literature, software must be made available to editors and reviewers. We strongly encourage code deposition in a community repository (e.g. GitHub). See the Nature Portfolio guidelines for submitting code & software for further information.

Data

Policy information about availability of data

All manuscripts must include a data availability statement. This statement should provide the following information, where applicable:

- Accession codes, unique identifiers, or web links for publicly available datasets
- A description of any restrictions on data availability
- For clinical datasets or third party data, please ensure that the statement adheres to our policy

All LC-MS data used in this paper are publicly available at the GNPS-MassIVE repository under the accession number MSV000087007. The raw data from the bottom-up proteomics analysis are publicly available at the GNPS-MassIVE repository under the MassIVE accession number MSV000087027. The raw-reads of RNA sequencing were deposited at the Sequencing Read Archive in the NCBI (National Center for Biotechnology Information) database under accession number PRJNA706894. The protein structures of MasL, MasL-collimonin C and MasL-collimonin D complex were deposited at PDB database under code 7EI3, 7EI4 and 7FEA.

Field-specific reporting

Please select the one below that is the best fit for your research. If you are not sure, read the appropriate sections before making your selection.

☒ Life sciences ☐ Behavioural & social sciences ☐ Ecological, evolutionary & environmental sciences

For a reference copy of the document with all sections, see nature.com/documents/nr-reporting-summary-flat.pdf

Life sciences study design

All studies must disclose on these points even when the disclosure is negative.

Sample size	Three independent biological replicates for minimum inhibitory concentration determination and genetic rescue and Enzymatic inhibition assays.
Data exclusions	Any data was not excluded from consideration.
Replication	The results in this study were consistently replicated across multiple experiments, and all replicates exhibited similar results.
Randomization	Randomization is not relevant to our study. The culture of microorganisms and subsequent measurements of the lag time distributions were performed under uniform physical conditions. Thus, it is possible to compare different experiments.
Blinding	The blinding was not necessary in this study, because the results of biological replicates of all experiments are consistent and no additional judgment or interpretation is required.

Reporting for specific materials, systems and methods

We require information from authors about some types of materials, experimental systems and methods used in many studies. Here, indicate whether each material, system or method listed is relevant to your study. If you are not sure if a list item applies to your research, read the appropriate section before selecting a response.

Materials & experimental systems

n/a	Involved in the study	
☒	☐	Antibodies
☐	☒	Eukaryotic cell lines
☒	☐	Palaeontology and archaeology
☒	☐	Animals and other organisms
☒	☐	Human research participants
☒	☐	Clinical data
☒	☐	Dual use research of concern

Methods

n/a	Involved in the study	
☒	☐	ChIP-seq
☒	☐	Flow cytometry
☒	☐	MRI-based neuroimaging

Eukaryotic cell lines

Policy information about cell lines

Cell line source(s)	Candida albicans ATCC18804
Authentication	Bioresource Collection and Research Center (BCRC), Food Industry Research and Development Institute, Taiwan

Mycoplasma contamination	The cell line was not tested for mycoplasma contamination.
Commonly misidentified lines (See ICLAC register)	Nil.
